# Structural basis of TRPA1 inhibition by HC-030031 utilizing species-specific differences

**DOI:** 10.1038/srep37460

**Published:** 2016-11-22

**Authors:** Rupali Gupta, Shigeru Saito, Yoshiharu Mori, Satoru G. Itoh, Hisashi Okumura, Makoto Tominaga

**Affiliations:** 1 Division of Cell Signaling, Okazaki Institute for Integrative Bioscience (National Institute for Physiological Sciences), National Institutes of Natural Sciences, Okazaki, Japan; 2 Department of Physiological Sciences, SOKENDAI (The Graduate University for Advanced Studies), Okazaki, Japan; 3 Research Center for Computational Science, Institute for Molecular Science, Okazaki, Japan; 4 Department of Structural Molecular Science, SOKENDAI (The Graduate University for Advanced Studies), Okazaki, Japan

## Abstract

Pain is a harmful sensation that arises from noxious stimuli. Transient receptor potential ankyrin 1 (TRPA1) is one target for studying pain mechanisms. TRPA1 is activated by various stimuli such as noxious cold, pungent natural products and environmental irritants. Since TRPA1 is an attractive target for pain therapy, a few TRPA1 antagonists have been developed and some function as analgesic agents. The responses of TRPA1 to agonists and antagonists vary among species and these species differences have been utilized to identify the structural basis of activation and inhibition mechanisms. The TRPA1 antagonist HC-030031 (HC) failed to inhibit frog TRPA1 (fTRPA1) and zebrafish TRPA1 activity induced by cinnamaldehyde (CA), but did inhibit human TRPA1 (hTRPA1) in a heterologous expression system. Chimeric studies between fTRPA1 and hTRPA1, as well as analyses using point mutants, revealed that a single amino acid residue (N855 in hTRPA1) significantly contributes to the inhibitory action of HC. Moreover, the N855 residue and the C-terminus region exhibited synergistic effects on the inhibition by HC. Molecular dynamics simulation suggested that HC stably binds to hTRPA1-N855. These findings provide novel insights into the structure-function relationship of TRPA1 and could lead to the development of more effective analgesics targeted to TRPA1.

Pain usually arises from noxious stimuli and alerts us to potential danger, as well aids in the avoidance of similar experiences in the future. Considerable advances have been made over the last two decades in our understanding of peripheral pain mechanisms and the development of new analgesics. Mounting evidence suggests an important role in acute, inflammatory and chronic pain states for a subset of transient receptor potential (TRP) ion channels. TRP channels are nonselective cation channels that form a superfamily based on their structural similarity; this includes a six transmembrane (TM) domain with a pore region between TM5 and TM6^1^. Among them, TRPA1, a member of TRPA subfamily, is one of the targets for studying pain mechanisms. TRPA1 is known to be activated by various nociceptive stimuli such as noxious cold (potentially in rodents), pungent natural products like cinnamaldehyde, and environmental irritants like acrolein^2^. Moreover, TRPA1 is predominantly expressed in nociceptive neurons in the dorsal root ganglion, trigeminal ganglion and nodose ganglion^3^. To date, a few TRPA1 antagonists have been developed and entered into pre-clinical trials^4^. The discovery of selective TRPA1 antagonists has allowed studies to address the role of TRPA1 in health (as a potential drug target for pain relief) as well as in various animal disease models^5^,^6^.

Given that TRPA1 is a crucial nociceptive receptor, it is widely conserved among species. Characterization of TRPA1 from various species revealed that the sensitivity to different antagonists is species-specific because selective TRPA1 antagonists have been developed using mammalian TRPA1 (Fig. S1)^7^. Due to this species diversity, comparative analysis of TRPA1 among different species has proven informative for understanding structure-function relationships^8^,^9^. For example, A967079 (A96) and AP18, both of which are structurally similar, are potent mammalian TRPA1 antagonists, although their inhibitory effects on TRPA1 vary among species^10^,^11^,^12^. Comparative and mutagenesis experiments with TRPA1 from different species revealed that several amino acids in the TM5 domain are crucial to the effects of these two antagonists^9^,^12^,^13^. Furthermore, a recent study reporting the detailed structure of human TRPA1 identified a binding site for A96 that is in the vicinity of sites found previously^14^. Therefore, investigation of the pharmacology of TRPA1 antagonists in different species will provide clues for identifying the structural basis of inhibition^7^.

Apart from A96, previous research^14^ failed to identify an action site for HC-030031 (HC), another potent mammalian TRPA1 antagonist^15^,^16^, thus there are no reports on the inhibitory mechanism on TRPA1 by HC, which is structurally different from A96 or AP18. The inhibitory effect of HC also differs among species. While HC inhibits TRPA1 from green anole and chicken, it fails to inhibit western clawed frog TRPA1 (fTRPA1) in a heterologous expression system^10^,^12^. Therefore, we attempted to identify amino acid residues (or regions) involved in the inhibitory effects of HC in order to understand the molecular mechanism of TRPA1 inhibition. In the present study, we utilized species-specific differences in HC inhibition to show that a single amino acid residue in the linker region of TM4 and TM5 is an important residue for the antagonistic action of HC. In addition, molecular dynamics simulation using hTRPA1 suggested that this single amino acid potentially binds to HC through hydrogen bonding. We also showed that this single amino acid synergistically interacts with the C-terminal region to enhance TRPA1 inhibition. By utilizing species differences, these findings can aid in understanding the structure-function relationship of TRPA1 and provide novel insight into the search for new analgesics targeting TRPA1.

## Results

### Antagonistic activity of HC differs between human and frog TRPA1

**Figure 1:**
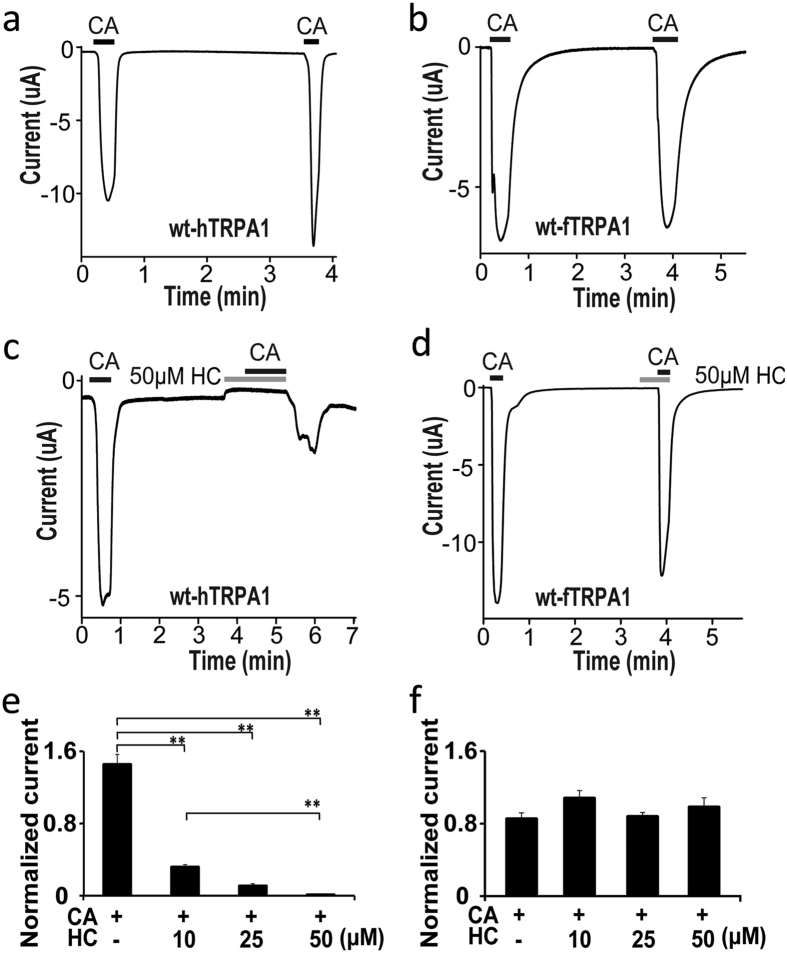
Species-specific differences in HC antagonism. (**a,b**) Representative traces of wt-hTRPA1 (**a**) or wt-fTRPA1 (**b**) currents in response to repeated CA application in *X. laevis* oocytes. (**c,d**) Representative traces showing that 50 μM HC inhibited CA-evoked currents in wt-hTRPA1 (**c**), but not in wt-fTRPA1 (**d**). (**e,f**) The differences in the inhibitory effects of HC (10, 25 and 50 μM) on CA-induced currents in oocytes expressing wt-hTRPA1 (**e**) or wt-fTRPA1 (**f**). Normalized current: ratio of the current amplitude in the second CA stimulation to that of the first with or without HC. (**P < 0.01), one way ANOVA post hoc Tukey test. Data are shown as the mean ± SEM. (**e**) wt-hTRPA1 (CA, n = 17; CA + 10 μM HC, n = 20; CA + 25 μM HC, n = 18; CA + 50 μM HC, n = 17). (f) wt-fTRPA1 (CA, n = 12; CA + 10 μM HC, n = 7; CA + 25 μM HC, n = 13; CA + 50 μM HC, n = 14).

In order to compare the antagonistic effects of HC on TRPA1 between human and frog, we first used a two-electrode voltage-clamp method to examine the responses of TRPA1 to its agonist cinnamaldehyde (CA) in *X. laevis* oocytes. Since CA-evoked responses were relatively fast and reversible^17^ compared with allyl isotiocyanate or carvacrol (Fig. S2d,e). CA concentrations higher than the reported EC_50_ values (0.13 mM and 0.39 mM for hTRPA1 and fTRPA1, respectively) were chosen^10^,^13^, and we waited for the CA-evoked currents to be stabilized or desensitized. Figure 1a and b shows the reversible current responses of wild type hTRPA1 (wt-hTRPA1) and fTRPA1 (wt-fTRPA1) to repeated application of 0.3 and 0.5 mM CA, respectively. TRPA1-mediated currents were activated by CA with approximately similar amplitude for the first and second stimulation, although the second CA (0.3 mM) evoked wt-hTRPA1 currents that were sometimes larger than the first stimulation (Fig. 1a). The CA-evoked TRPA1 currents showed desensitization after reaching the peak and returned to baseline levels upon CA washout in *X. laevis* oocytes expressing either wt-hTRPA1 or wt-fTRPA1 as previously reported for wt-hTRPA1^18^. Next, we applied HC along with CA during the second application to observe its antagonistic properties (Fig. 1c). Simultaneous application of HC and CA completely suppressed the CA-activated wt-hTRPA1 currents in *X. laevis* oocytes in a dose dependent manner (Fig. 1c,e), while we observed some current activation after washout probably because washout effects of covalent modification by CA take longer to appear than those of HC washout. On the other hand, HC failed to suppress CA-activated wt-fTRPA1 currents at any of the concentrations used (Fig. 1d,f). Although similar dose-dependent inhibition by HC was observed for CA (0.1 mM)-evoked wt-hTRPA1 (Fig. S2a), we used 0.3 mM CA for activation of wt-hTRPA1 in the rest of the experiments since 0.1 mM CA is lower than the reported EC_50_ value^13^. Performing the experiments in the absence of extracellular Ca^2+^ would avoid reported effects of Ca^2+^ on TRPA1 channel properties^19^,^20^,^21^. However, we decided to carry out the experiments in the presence of extracellular Ca^2+^ because channel activation by CA and inactivation upon CA washout were both very slow in the absence of extracellular Ca^2+^ (Fig. S2b,c)^22^,^23^, which makes the analysis of inhibition on the second application impossible. We also observed the species difference of HC sensitivity for hTRPA1 and fTRPA1 currents activated by the non-electrophilic compound carvacrol (Fig. S2d,e), suggesting that the structural basis for HC-induced inhibition is independent of the activation mechanism. We thus decided to focus on the CA-evoked TRPA1 currents.

### A 5 and 6 transmembrane domain region is involved in HC-induced inhibition

**Figure 2:**
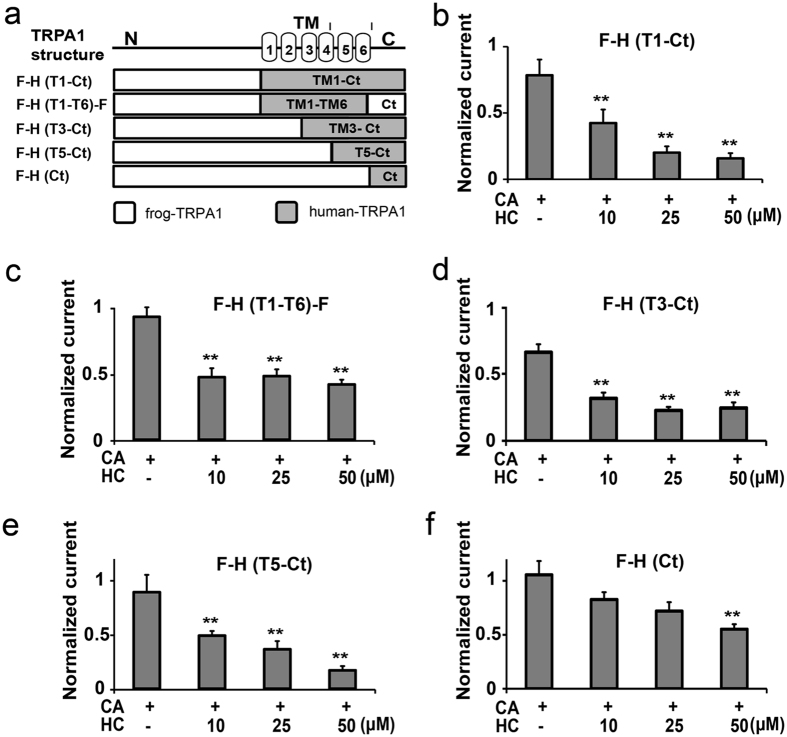
Effects of HC on various chimeric fTRPA1 and hTRPA1 channels. (**a**) Schematic representation of the hTRPA1 and fTRPA1 chimeras. hTRPA1 and fTRPA1 are depicted in grey and white, respectively. The regions of hTRPA1 included in the chimeras are indicated by brackets. (**b–f**) The effects of HC at different concentrations on the CA-evoked currents. Normalized current: ratio of the current amplitude in the second CA stimulation to that of the first with or without HC. (**P < 0.01), one way ANOVA post hoc Tukey test. Data are shown as the mean ± SEM. (**b**) F-H (T1-Ct) (0.3 mM CA, n = 11; CA + 10 μM HC, n = 9; CA + 25 μM HC, n = 9; CA + 50 μM HC, n = 10). (**c**) F-H (T1-T6)-F (0.5 mM CA, n = 16; CA + 10 μM HC, n = 6; CA + 25 μM HC, n = 12; CA + 50 μM HC, n = 11). (**d**) F-H (T3-Ct) (0.5 mM CA, n = 15; CA + 10 μM HC, n = 5; CA + 25 μM HC, n = 10; CA + 50 μM HC, n = 8). (**e**) F-H (T5-Ct) (0.2 mM CA, n = 7; CA + 10 μM HC, n = 5; CA + 25 μM HC, n = 7; CA + 50 μM HC, n = 8). (**f**) F-H (Ct) (0.1 mM CA, n = 12; CA + 10 μM HC, n = 5; CA + 25 μM HC, n = 5; CA + 50 μM HC, n = 13).

In order to narrow down the region responsible for the antagonistic effects of HC, chimeric hTRPA1 and fTRPA1 channels were constructed and their channel properties were examined in *X. laevis* oocytes. We generated several chimeric combinations between hTRPA1 and fTRPA1 by introducing the N-terminus, different TM domains, and the C-terminus of fTRPA1 into hTRPA1 (Fig. 2a). We used the following criteria for determining the CA concentrations for each chimera by examining the dose-dependent profiles (Fig. S3): (1) the concentration is higher than the EC_50_ values; (2) the CA concentration evokes similar current amplitudes during the first and second application.

The N-terminus of hTRPA1 was first replaced with that of fTRPA1 [F-H (T1-Ct)], and HC significantly suppressed the CA-evoked currents in this chimera (Figs 2b and S4a). The next chimera was made by switching the N and C-termini of hTRPA1 with those of fTRPA1 [F-H (T1-T6)-F], and HC significantly inhibited the CA-activated currents in this chimera even at 10 μM, although we did not observe clear dose-dependency (Figs 2c and S4b). These results indicated that the major HC antagonistic site(s) of action are within the TM domains.

To further narrow down the region within the TM domains, F-H (T3-Ct) and F-H (T5-Ct) chimeras were constructed (Fig. 2a), and these were also apparently inhibited by HC (Figs 2d,e and S4c,d) similar to wt-hTRPA1. These results indicated that a region containing TM5 to TM6 plays a major role in TRPA1 inhibition by HC. We also examined the involvement of the C-terminus by replacing the fTRPA1 C-terminus with that of hTRPA1 [F-H (Ct)]. This chimera showed weak inhibition by HC in spite of its high sensitivity to CA (Fig. S4e). Significant inhibition of chimeric channel activity by HC only occurred at 50 μM (Fig. 2f), confirming the greater involvement of TM5 and TM6 in HC-induced inhibition. Thus, we decided to focus on the TM5 and TM6 domains to identify the specific regions involved in the inhibition by HC.

### A single amino acid residue between TM4-TM5 in hTRPA1 is responsible for the antagonistic effects of HC

**Figure 3:**
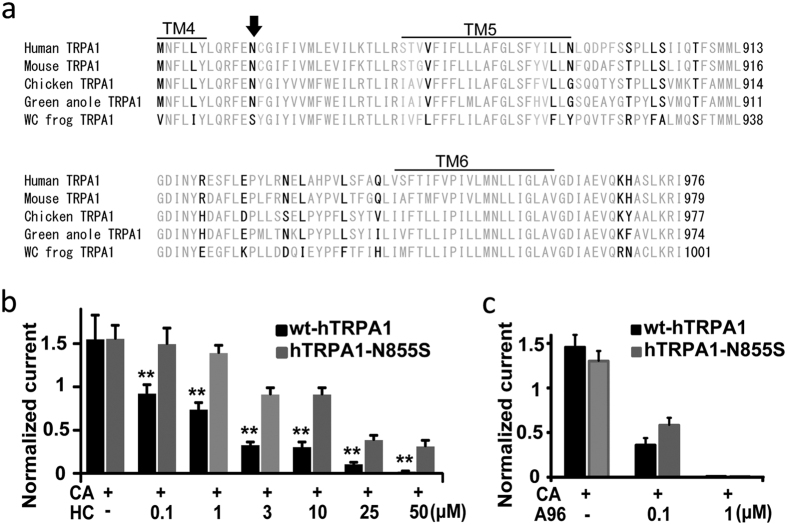
Identification of the amino acid responsible for the inhibitory effects of HC on hTRPA1. (**a**) Amino acid alignment of the regions delineated by the chimeric analysis shown in Fig. 2. Amino acids were examined using the following formula: (human TRPA1 ≈ mouse TRPA1 ≈ green anole TRPA1 ≈ chicken TRPA1) ≠ (western clawed (WC) frog TRPA1); these differences are highlighted in black. The arrow indicates the amino acid involved in the inhibitory effect of HC. (**b–d**) Differences in the inhibitory effects of HC (**b** and **c**) or A96 (**d**) at different concentrations on CA-evoked currents in wt-hTRPA1 or hTRPA1-N855S. Normalized current: ratio of the current amplitude in the second CA stimulation to that of the first with or without HC. Each bar represents the mean ± SEM. (**P < 0.01), one way ANOVA post hoc Tukey test or t-test. (**b**) wt-hTRPA1 and hTRPA1-N855S (0.3 mM CA, n = 5,5; CA + 0.1 μM HC, n = 6,6; CA + 1 μM HC, n = 7,6; CA + 3 μM HC, n = 6,6; CA + 10 μM HC, n = 6,6; CA + 25 μM HC, n = 6,7; CA + 50 μM HC, n = 5,7). (**c**) wt-hTRPA1 and hTRPA1-N855S (0.3 mM CA, n = 11,11; CA + 0.1 μM A96, n = 4,5; CA + 1 μM A96, n = 4,5).

We searched for candidate amino acid residue(s) involved in the antagonistic activity of HC by comparing the amino acid sequence of TRPA1 from 5 different species within the region identified by the chimeric analyses above (Fig. 2). We examined only those amino acids that are conserved (or similar) among TRPA1 from humans, mice, chickens, and green anoles, but are different in fTRPA1 since fTRPA1 is insensitive to HC, while TRPA1 from the other species is inhibited by HC^10^,^11^. We hypothesized that these amino acid(s) might be involved in the antagonistic activity of HC. All of the potential amino acid candidates are shown in Fig. 3a. To verify this hypothesis, we constructed hTRPA1 point mutants containing single or double amino acid substitutions in which the amino acids were changed to the residue(s) found in fTRPA1. The inhibitory effects of 50 μM HC were examined in all hTRPA1 mutants and most of them showed nearly complete inhibition except for a single mutant, hTRPA1-N855S (Supplementary Table S1).

Next, we examined the detailed channel properties of the hTRPA1-N855S mutant in side-by-side comparisons with wt-hTRPA1 in the same preparations. CA (0.3 mM)- evoked currents for the first and second stimulation had an approximately similar size in *X. laevis* oocytes expressing either wt-hTRPA1 or hTRPA1-N855S (Figs 1a and S5a). Simultaneous application of 50 μM HC with CA completely suppressed CA-evoked wt-hTRPA1 activation in *X. laevis* oocytes (Fig. 1c) while hTRPA1-N855S showed less susceptibility to 50 μM HC (Fig. S5b). We always observed the transient activation of hTRPA1-N855S by CA in the presence of 50 μM HC that was probably due to the reduction in the HC-sensitivity of the mutant, as we saw for wt-hTRPA1 currents in the presence of lower concentrations of HC (data not shown). Although HC suppressed CA-evoked activity of both wt-hTRPA1 and hTRPA1-N855S in a dose-dependent manner (Figs 1e and 3b), hTRPA1-N855S showed significantly less sensitivity to HC than wt-hTRPA1 at all concentrations examined (Fig. 3b). In contrast, simultaneous application of A96 (0.1 and 1 μM) along with CA suppressed activity of CA-evoked currents in *X. laevis* oocytes expressing either wt-hTRPA1 or hTRPA1-N855S (Figs 3c and S5c,d) to nearly the same extent, indicating that N855 in hTRPA1 is specifically involved in HC-induced inhibition.

**Figure 4:**
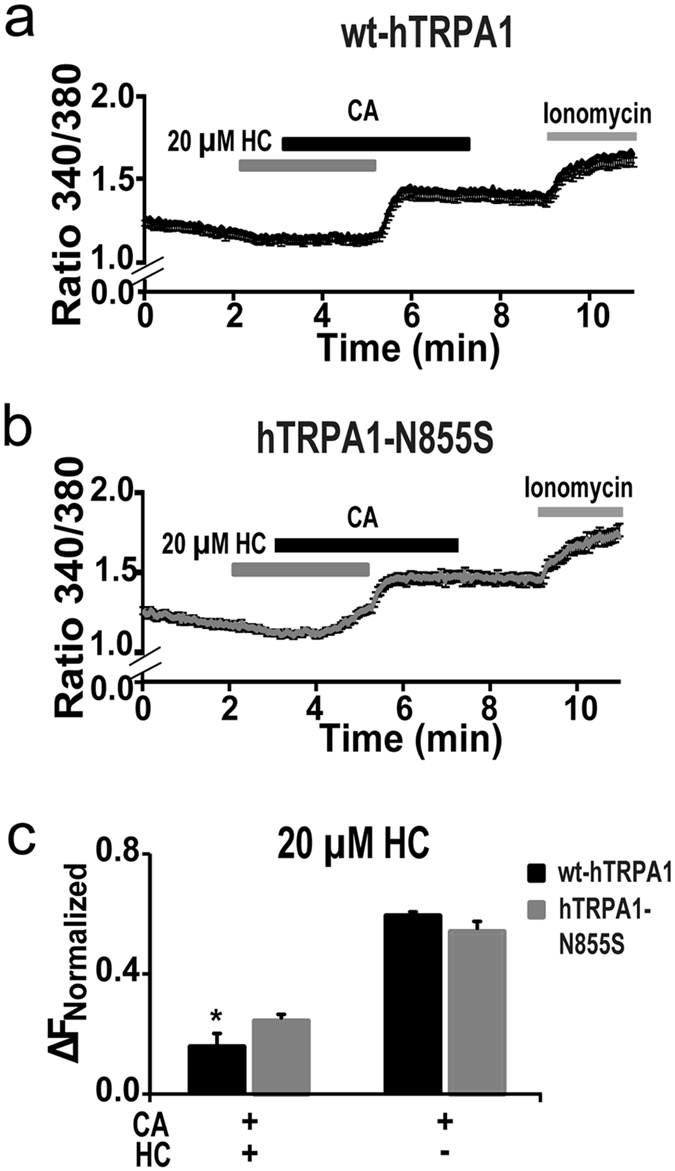
Effects of HC on the hTRPA1-N855S mutant expressed in HEK293T cells. (**a,b**) The effects of HC (20 μM) on the CA (0.1 mM)-evoked increase in [Ca^2+^]_*i*_ in HEK293 cells expressing wt-hTRPA1 or hTRPA1-N855S. Each bar represents the mean ± SEM. (*P < 0.05), t-test. (**c**) Comparison of the normalized ratio to the ionomycin responses between wt-hTRPA1 (n = 5 with and 5 without HC, respectively) and hTRPA1-N855S (n = 7 with and 7 without HC, respectively).

The above observations were further confirmed using a different expression system. CA stimulation increased [Ca^2+^]_*i*_ in a dose-dependent manner in HEK293T cells expressing either wt-hTRPA1 or hTRPA1-N855S (Fig. S6a,b) with apparently higher sensitivity than in *X. laevis* oocytes^13^. Because it is difficult to wash out CA from HEK293T cells, a single CA application was used and the responses were normalized to the ionomycin responses. While CA increased [Ca^2+^]_*i*_ to a similar extent for both WT and mutant channel (Fig. S6c–e), 20 μM HC showed significantly less inhibition of CA-induced [Ca^2+^]_*i*_ influxes in hTRPA1-N855S compared to wt-hTRPA1 (Fig. 4a–c). These results suggested that N855 in hTRPA1 is important for the antagonistic activity of HC.

**Figure 5:**
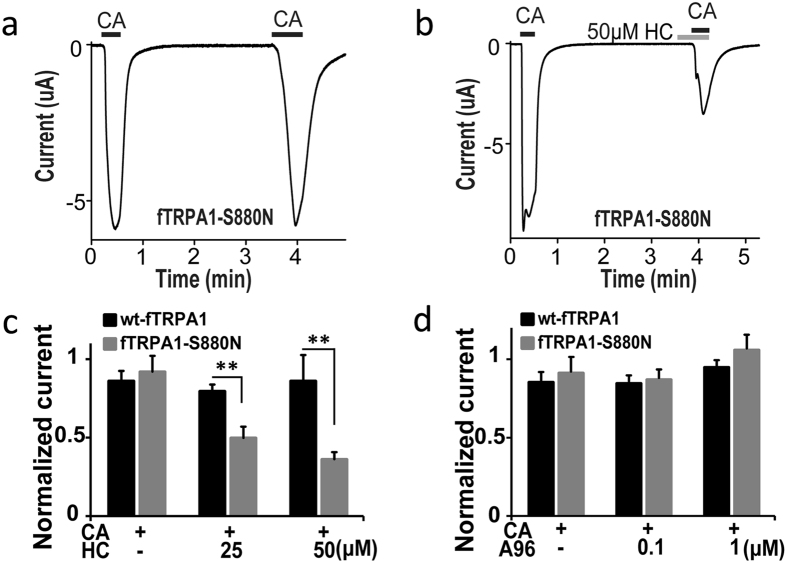
HC inhibitory effects on fTRPA1 mutants. (**a,b**) Representative traces of CA (0.5 mM)-evoked currents in *X. laevis* oocytes expressing fTRPA1-S880N without (**a**) or with (**b**) HC (50 μM). (**c**,**d**) The effects of HC (**c**) or A96 (**d**) at different concentrations on CA-evoked currents in wt-fTRPA1 and fTRPA1-S880N. Normalized current: ratio of the current amplitude in the second CA stimulation to that of the first with or without HC. Each bar represents the mean ± SEM. (**P < 0.01), t-test. (**c**) (CA, n = 12,11; CA + 25 μM HC, n = 5,6; CA + 50 μM HC, n = 7,9). (**d**) (CA, n = 12,11; CA + 0.1 μM A96, n = 6,5; CA + 1 μM A96, n = 5,7).

To confirm the importance of N855 in hTRPA1 on the effects of HC, we examined whether reverse mutation at a corresponding position in fTRPA1 alters the effects of HC. The serine at position 880 in fTRPA1 (corresponding to 855 in hTRPA1) was replaced with asparagine (fTRPA1-S880N). Although CA (0.5 mM) evoked similar sized currents in the first and second stimulation in *X. laevis* oocytes expressing either wt-fTRPA1 or fTRPA1-S880N (Figs 1b and 5a), HC reduced the CA-evoked currents in the second application in fTRPA1-S880N in a dose-dependent manner, but not in wt-fTRPA1 (Figs 1b and 5b,c). These results further support the importance of the asparagine residue at this position, and demonstrate its specificity for HC since the effects of A96 on fTRPA1-S880N remained unaffected (Fig. 5d).

### Inhibitory effects of HC on zebrafish TRPA1

**Figure 6:**
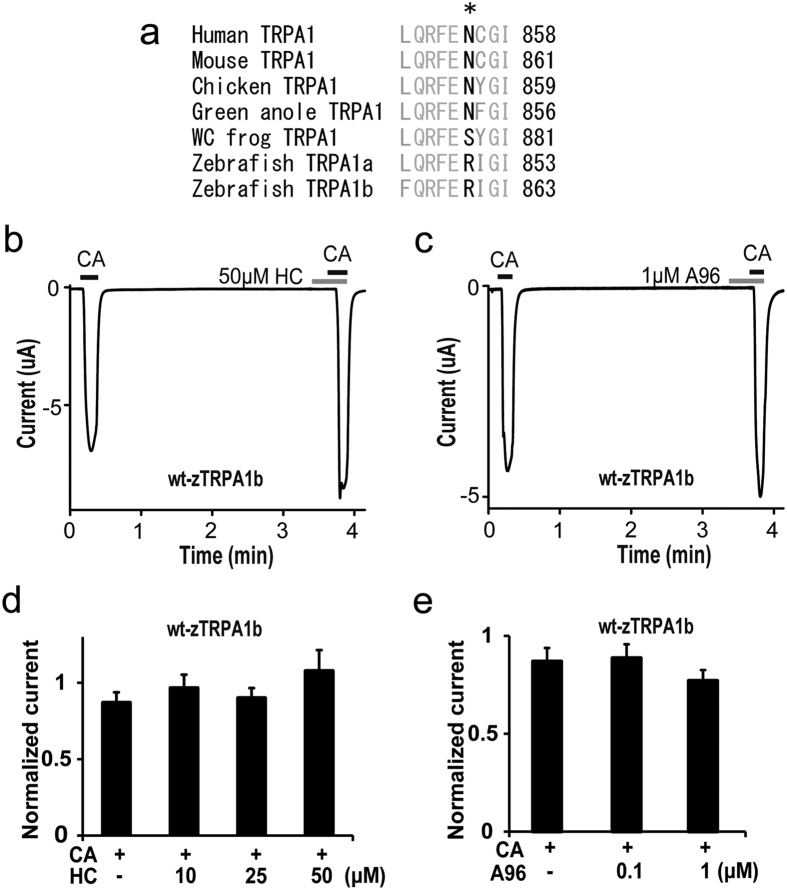
Effects of TRPA1 antagonists on zTRPA1b. (**a**) Comparison of N855 in hTRPA1 (asterisk) with that in mouse, chicken, green anole, western clawed (WC) frog, and zebrafish TRPA1. (**b**,**c**) Representative traces of CA-evoked currents in wt-zTRPA1b in the presence of 50 μM HC (**b**) or 1 μM A96 (**c**). (**d**,**e**) Effects of HC (**d**) or A96 (**e**) at different concentrations on CA (1 mM)-evoked wt-zTRPA1b currents. Normalized current: ratio of the current amplitude in the second CA stimulation to that of the first with or without HC. Each bar represents the mean ± SEM. (**d**) (CA, n = 18; CA + 10 μM HC, n = 11; CA + 25 μM HC, n = 11; CA + 50 μM HC, n = 13). (**e**) (CA, n = 18; CA + 0.1 μM A96, n = 10; CA + 1 μM A96, n = 10).

To further confirm the importance of the asparagine residue on TRPA1 inhibition by HC, we examined the effect of HC on zebrafish TRPA1 since the corresponding residue is arginine (not asparagine) in wild type zebrafish (wt-z) TRPA1a and TRPA1b (Fig. 6a). To date, there have been no reports on the effects of mammalian TRPA1 antagonists on wt-zTRPA1. Therefore, we first examined the responses of both wt-zTRPA1a and wt-zTRPA1b to HC and A96. Because wt-zTRPA1a and wt-zTRPA1b exhibited different sensitivities to CA (Fig. S7a,b), different concentrations of CA were used (0.3 mM for wt-zTRPA1a and 1 mM for zTRPA1b). wt-zTRPA1a showed desensitization in the second CA application even at low concentrations (Fig. S7c) while wt-zTRPA1b exhibited similar CA-activated currents in both stimulations (Fig. S7d). Therefore, we proceeded with wt-zTRPA1b for further analysis and found that neither HC nor A96 had any inhibitory effects on wt-zTRPA1b even at high concentrations in *X. laevis* oocytes (Fig. 6b–e).

**Figure 7:**
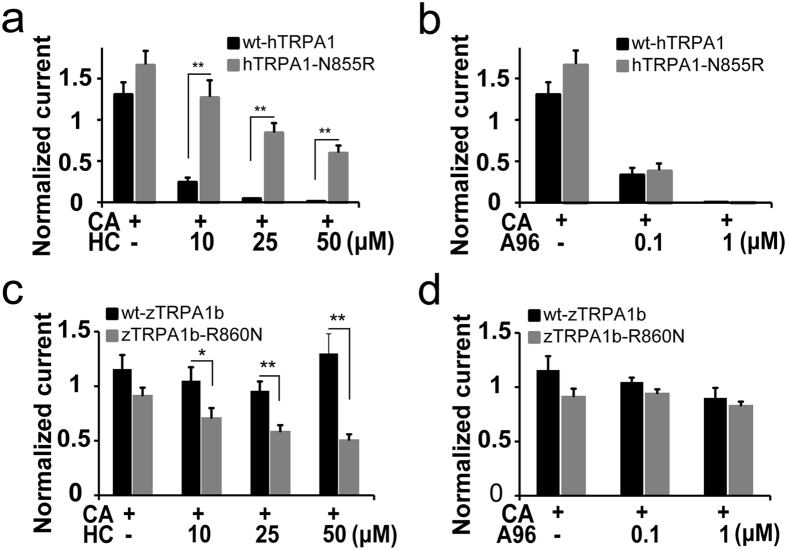
Changes in the inhibitory effects of HC by a single point mutation in hTRPA1 and zTRPA1b. (**a–d**) Effects of HC (**a,c**) or A96 (**b,d**) at different concentrations on CA-evoked currents in wt-hTRPA1 and hTRPA1-N855R (**a,b**) or on the CA-evoked currents in wt-zTRPA1b and zTRPA1b-R860N (**c,d**). Normalized current: ratio of the current amplitude in the second CA stimulation to that of the first with or without HC. Each bar represents the mean ± SEM. (**P < 0.01, *P < 0.05), t-test. (**a**) wt-hTRPA1 and hTRPA1-N855R (0.3 mM CA, n = 9,9; CA + 25 μM HC, n = 8,8; CA + 25 μM HC, n = 7,8; CA + 50 μM HC, n = 7,8). (**b**) wt-hTRPA1 and hTRPA1-N855R (0.3 mM CA, n = 9,9; CA + 0.1 μM A96, n = 4,5; CA + 1 μM A96, n = 4,4). (**c**) wt-zTRPA1b and zTRPA1b-R860N (1 mM CA, n = 5,6; CA + 10 μM HC, n = 5,6; CA + 25 μM HC, n = 5,6; CA + 50 μM HC, n = 5,6). (**d**) wt-zTRPA1b and zTRPA1b-R860N (1 mM CA, n = 5,6; CA + 0.1 μM A96, n = 4,4; CA + 1 μM A96, n = 4,4).

In order to confirm the importance of arginine, we replaced the asparagine of hTRPA1 at position 855 to the arginine (hTRPA1-N855R) found in wt-zTRPA1b at the corresponding position. While CA (0.3 mM) evoked similar sized currents in the first and second stimulation in *X. laevis* oocytes expressing either wt-hTRPA1 or hTRPA1-N855R (Figs 1a and S8a), hTRPA1-N855R exhibited reduced inhibitory effects by simultaneous application of 50 μM HC (Fig. S8c) which completely suppressed the CA-evoked wt-hTRPA1 currents (Fig. 1c). We observed transient hTRPA1-N855R currents evoked by CA in the presence of HC (Fig. S8c), a phenomenon similar to the hTRPA1-N855S-mediated currents (Fig. S5b) that may be due to reduced HC sensitivity. Although HC suppressed the CA-evoked activity of both hTRPA1 and hTRPA1-N855R in a dose-dependent manner, hTRPA1-N855R showed significantly less inhibition by HC (Fig. 7a). In contrast, the inhibitory effect of A96 remained unaffected in hTRPA1-N855R (Figs 7b and S8e), indicating that the effect of the mutation at position 855 in hTRPA1 is specific to HC.

We next examined the effect of HC in a reverse mutant by replacing arginine at position 860 in wt-zTRPA1b with asparagine (zTRPA1-R860N). We examined the effects of HC along with CA in the second application after confirming that repeated applications exhibited similar current activity (Fig. S8b). Concurrent application of HC with CA in the second application led to significant reduction in current amplitudes in zTRPA1-R860N although wt-zTRPA1b showed no inhibition (Figs 7c and S8d). As expected, this effect was specific to HC activity, as A96 failed to inhibit either wt-zTRPA1b or zTRPA1b-R860N currents (Figs 6c, 7d and S8f). These results indicated that the single amino acid residue located in the linker region between TM4 and TM5 plays an important role in the antagonistic effects of HC.

### Synergistic effects between N855 and the C-terminus for complete HC inhibition of hTRPA1

**Figure 8:**
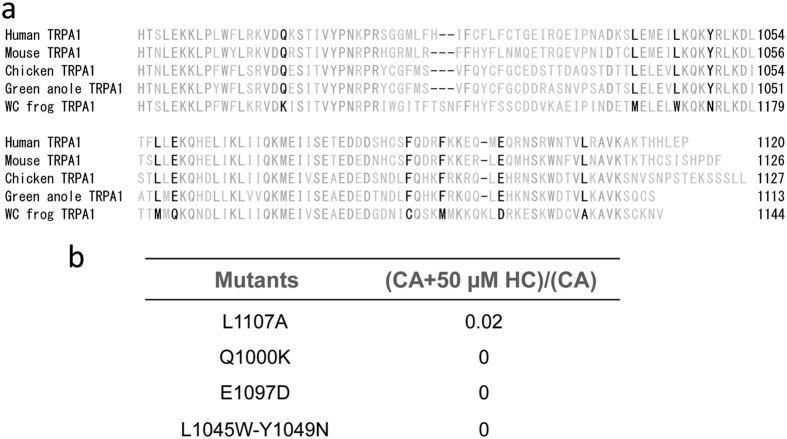
Effects of HC on point mutations in the C-terminal region of hTRPA1. (**a**) Amino acid alignment of the C-terminal regions of TRPA1 from different species. Amino acids were evaluated using the following formula: (human TRPA1 ≈ mouse TRPA1 ≈ green anole TRPA1 ≈ chicken TRPA1) ≠ (western clawed (WC) frog TRPA1); these differences are highlighted in black. (**b**) Effects of 50 μM HC on CA-evoked currents. Average normalized currents for CA with 50 μM HC were divided by the average normalized current for CA alone. For each mutant, n = 3–5.

Given that we failed to observe complete attenuation of the HC effects in any of the above hTRPA1 point mutants, we searched for another action site of HC activity. Since a high concentration of HC exhibited significant inhibition of the F-H (Ct) chimera (Fig. 2f), we performed point mutant analysis within the C-terminus. We made several mutants for potential amino acid positions which satisfied the criteria previously described. All potential amino acid candidates that satisfy the above formula are shown in Fig. 8a. We constructed hTRPA1 mutants containing single or double amino acid substitutions in which the amino acids were changed to residues found in fTRPA1. Unfortunately, all of the mutants which we successfully made showed complete inhibition by HC (Fig. 8b).

**Figure 9:**
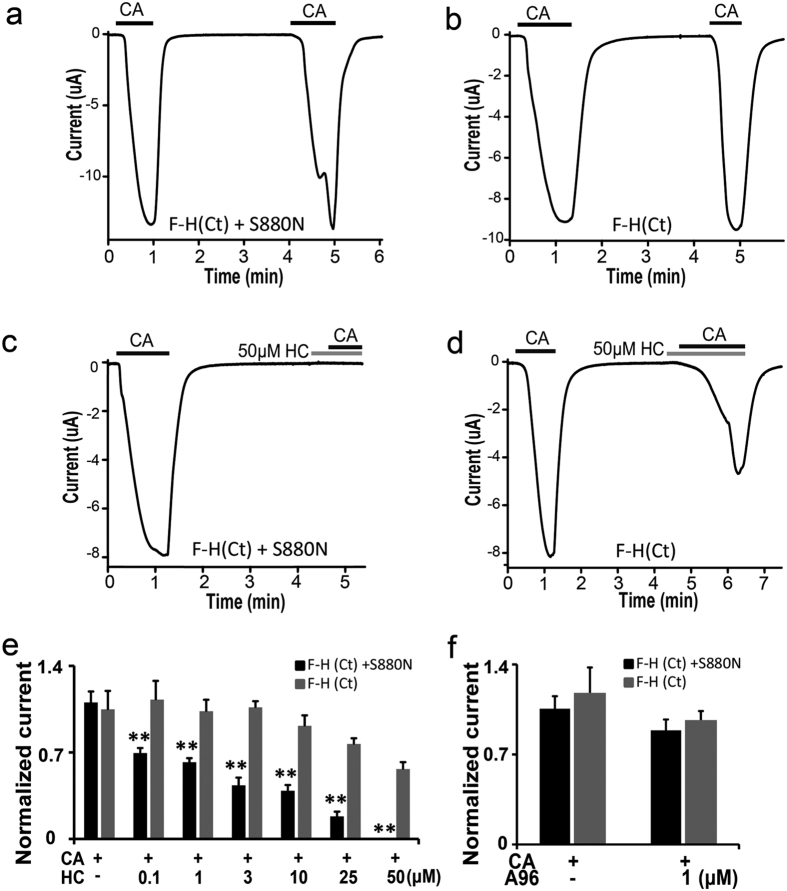
Synergistic interaction between N855 and the C-terminus of TRPA1 on the inhibitory effects of HC. (**a,b**) Representative traces of CA (0.1 mM)-evoked currents in X. laevis oocytes expressing the F-H (Ct) chimera with the fTRPA1-S880N mutation [F-H (Ct) + S880N] (**a**) or the F-H (Ct) chimera alone (**b**). (**c,d**) Effects of 50 μM HC on CA-evoked currents in the F-H (Ct) + S880N (**a**) or F-H (Ct) chimera alone (**b**). (**e,f**) Effects of HC (**c**) or A96 (**d**) at different concentrations on CA-evoked currents in the F-H (Ct) + S880N or F-H (Ct) chimera alone. Normalized current: ratio of the current amplitude in the second CA stimulation to that of the first with or without HC. Each bar represents the mean ± SEM. (**P < 0.01), t-test. (**e**) F-H (Ct) + fTRPA1-S880N and F-H (Ct) (0.1 mM CA, n = 12,12; CA + 0.1 μM HC, n = 6,4; CA + 1 μM HC, n = 5,5; CA + 3 μM HC, n = 6,4; CA + 10 μM HC, n = 9,8; CA + 25 μM HC, n = 9,9; CA + 50 μM HC, n = 9,9). (**f**) F-H (Ct) + fTRPA1-S880N and F-H (Ct) (0.1 mM CA, n = 8,8; CA + 1 μM A96, n = 3,3).

The high-resolution structure of hTRPA1 has now been made available and can provide a platform for understanding channel function^14^. We thus examined the amino acid at position N855 and found that it is near the C-terminal domain, suggesting the possibility that N855 interacts with the C-terminus. In order to demonstrate this, we introduced an 855-corresponding mutation into the F-H (Ct) chimera [F-H (Ct) + S880N]. This mutant was activated by CA application with a similar dose dependency as the F-H (Ct) chimera (Fig. 9a,b, data not shown). Surprisingly, F-H (Ct) + S880N showed complete and dose-dependent inhibition by HC, similar to wt-hTRPA1, compared to the F-H (Ct) chimera (Fig. 9c–e). As anticipated, this effect was specific for HC as A96 failed to inhibit either the F-H (Ct) + S880N or F-H (Ct) chimeras (Fig. 9f). Similar dose-dependent inhibition by HC of wt-hTRPA1 (Fig. S2a) and F-H (Ct) + S880N (Fig. 9e) suggested that the single amino acid residue located in the linker region between TM4 and TM5 displays synergistic effects with the human C-terminus resulting in complete inhibition of the TRPA1 channel by HC.

### Stable binding of HC and N855 in human TRPA1

**Figure 10:**
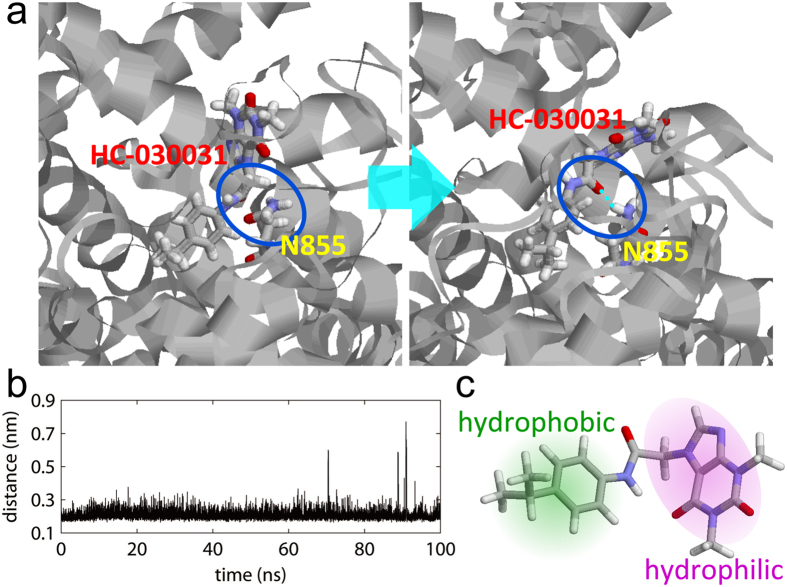
Molecular dynamics simulation confirming the stable bond between HC and N855. (**a**) Snapshots of the MD simulation at 0 ns (left) and at 100 ns (right). The HC molecule and amino acid residue N855 are shown in a stick representation. These snapshots were rendered using the RasMol graphic software^51^. (**b**) A time series of the distance between the O atom of HC and the H_δ_ atom of N855 in the MD simulation. (**c**) Highlighted hydrophobic (green) and hydrophilic (purple) regions of the HC molecule.

Lastly, we performed an MD simulation to investigate whether HC binds to N855 in human TRPA1 for 100 ns. Figure 10a shows the conformation of HC and N855 at 0 ns and 100 ns. A hydrogen bond was not formed between the O atom of the amide bond in HC and the H_δ_ atom in N855 at 0 ns, but it was formed immediately after the MD simulation began. This hydrogen bond was maintained during most of the simulation time. The definition of the secondary structure of the protein^24^ was used to determine if a hydrogen bond was formed or not. We calculated the time series of the distance between the O atom of HC and the H_δ_ atom of N855 as shown in Fig. 10b. This distance mostly fluctuated only about 0.2 nm, although the distance occasionally became longer than 0.2 nm. The hydrogen bond was broken at this time, but it was immediately formed again. This suggests that HC stably binds to N855. In addition, the hydrophobic portion of HC, as shown in Fig. 10c, was bound in the hydrophobic pocket near N855. This likely stabilizes the binding between HC and hTRPA1 (Movie 1).

We also performed an MD simulation for the N855S mutant of the hTRPA1. Figure S9a shows the conformation of HC and S855 at 100 ns. A hydrogen bond was not formed between HC and hTRPA1 in this conformation. As shown in Fig. S9b, the distance between the O atom of HC and the H_γ_ atom of S855 fluctuated between 0.2 nm and 0.5 nm. Although a hydrogen bond was sometimes formed for a short period, it was broken soon. A hydrogen bond was not formed stably in the N855S mutant.

## Discussion

Given the localization of TRPA1, a polymodal non-selective cation channel, to sensory nerve endings and its well established presence in pain and inflammation research, it has become an intriguing clinical drug target. Many research groups, including our own, have previously attempted to identify the binding mechanisms or the effective sites for potent mammalian TRPA1 antagonists. While the action sites of A96 and AP-18, which are structurally similar, were discovered^9^,^12^,^13^,^14^,^25^, those of HC remained undetermined. It is well documented that the effects of TRPA1 antagonists differ in a species-specific manner; therefore, we utilized these species-specific differences to identify the sites of the antagonistic activity of HC. In this study, we subjected TRPA1 from five different species (human, mouse, chicken, green anole, and frog) to sequence analysis which showed different HC sensitivity. Because TRPA1 is a unique polymodal signal detector whose activity varies in different species, it is important to expand the data to include new species in order to understand its distinct physiological roles. This is the first report to show the effects of A96 and HC on zTRPA1, which also verified the lack of inhibition by either antagonist (Fig. 6). We confirmed that HC failed to show any inhibitory effects on fTRPA1 or zTRPA1b (Figs 1 and 6) activated by CA in a heterologous expression system using *X. laevis* oocytes, whereas HC showed dose-dependent inhibition of hTRPA1 activity. Similar species-specific pharmacological activity of TRPA1 has been previously reported. Some examples include: (1) cysteine-attacking compound CMP1, activates rat TRPA1 but suppresses hTRPA1^26^; (2) menthol activates mouse TRPA1 at low concentrations but blocks it at high concentrations^9^; (3) molecules like AMG2504 and AMG7160 potently block hTRPA1 but activate rat TRPA1^27^; and (4) caffeine activates mouse TRPA1, but inhibits human TRPA1 via a single amino acid difference^28^. These species-specific differences in TRPA1 channel properties are the result of a few amino acid differences. Thus, small changes in amino acid sequence between different species could account for the differing TRPA1 sensitivities to specific agonists and antagonists.

Through chimeric and mutational studies with different species, we found that a single amino acid, N855, within the linker region of TM4 and TM5 is an important residue for the inhibition of hTRPA1 by HC. Mutants in which N855 in TRPA1 was replaced with serine or arginine, corresponding TRPA1 residues from frog and zebrafish, respectively, showed reduced HC sensitivity and reverse mutants (S880N and R860N), in which corresponding amino acids were changed to asparagine, led to increased HC sensitivity (Figs 3, 5 and 7). These results further support the importance of this amino acid in TRPA1 channel function. Interestingly, N855R showed less sensitivity towards HC than N855S, which could be partly explained by the positive charge of arginine and the observation of more stable inter-subunit salt bridges in N855R with amino acids at position 854 and 868 compared to N855S^29^.

Remarkably, a missense mutation in the same residue N855 to serine was previously shown to be associated with an autosomal-dominant heritable familial episodic pain syndrome triggered by cold, fasting and fatigue, resulting in upper body pain in humans^5^. Similar to that study, we failed to find any major difference in the EC_50_ value of CA between wt-hTRPA1 and hTRPA1-N855S mutants in heterologous expression systems using either *X. laevis* oocytes or HEK293T cells. As reported, the hTRPA1 mutant (N855S) associated with the familial episodic pain syndrome is a gain-of-function mutant^5^. This leads to the question, what is the correlation between the mutated amino acid associated with human disease and the amino acid changes observed in the fTRPA1 and zTRPA1b, both of which show insensitivity to HC? One possibility is that unknown endogenous TRPA1 inhibitors might act on the amino acid and mutations could thus lead to increased channel activity. Another possibility is that the functional changes observed in the N855S mutant may originate, at least in part, from changes to inter-subunit interactions between E854 and K868 in the proximity of N855, as proposed by Zíma *et al*.^29^. Our MD simulation results showed that HC forms a hydrogen-bond with N855 and the hydrophobic region of HC is surrounded by a hydrophobic pocket near N855 (Fig. 10), and this could suppress domain motion by preventing channel opening. One possible inhibitory mechanism is the localization of the HC binding position in the hinge between the domains (for example TM4 and TM5), and the hinge motion required for domain motion could be restricted by HC binding. Regardless of the relationship, these mutations or amino acid changes provide clues that may have enabled shifts in nociception over the course of evolution, while retaining chemical sensitivity in spite of the high degree of sequence differences across species.

Many groups, as reported above, have predicted the action sites of A96. The high-resolution 3D structure of hTRPA1 was recently published and showed an actual A96 binding site based on its unique density within a pocket formed by TM5 and TM6 and the first pore helix which is surrounded by the previously predicted sites. A96 could thus inhibit channel activity via an “induced fit” mechanism involving movement in nearby residues that comprise the pocket^14^. Moreover, HC could interfere with gating by binding to N855, or N855 could be indirectly involved in the inhibition by modulating the effects of HC.

While the single amino acid residue (N855 in hTRPA1) located in the linker region between TM4 and TM5 exhibited a partial but significant role in the effects of HC, the mutation of this amino acid together with the C-terminal replacement in fTRPA1 caused almost complete inhibition by HC (Fig. 9), similar to that observed in wt-hTRPA1. Since the 3D structure of hTRPA1 suggests that N855 is located just above the TRP-like domain in the C-terminus, and HC is a bigger molecule than A96, HC possibly interacts with the TRP-like domain to inhibit TRPA1 activity induced by CA. Alternatively, structural differences in the C-termini between fTRPA1 and hTRPA1 could indirectly change the structure of the HC binding pocket.

In the last decade, a few TRPA1 antagonists have been developed and entered into pre-clinical trials^4^. Some examples include: (1) A96 was reported to reduce pain-related behaviors in a model of osteoarthritis in rats^30^; (2) HC administration reduced cold hyperalgesia in rat^31^; and (3) HC and a TRPV1 antagonist, AMG 9810, could synergistically reduce pain-related behaviors in a pancreatitis model^32^. Therefore, TRPA1 antagonists are fascinating potential chemical compounds for pain relief. However, the structural basis of TRPA1 inhibition by HC remains to be elucidated, and would supply valuable information for understanding the inhibitory mechanisms of TRPA1. In the present study, we have explored the structural basis of TRPA1 inhibition by HC and identified N855 in the linker region between the TM4 and TM5 domains of hTRPA1 as an amino acid responsible for determining the sensitivity to HC antagonism. Substitution of this single amino acid affected the sensitivity of TRPA1 to HC in diverse species (hTRPA1, fTRPA1 and zTRPA1b). Future research should be aimed at confronting the challenges that have emerged from the species-specificity of HC, and the mechanisms by which TRPA1 antagonists directly affect channel affinity to drug binding or alter the structure by interrupting the entry pathway of the compounds. These studies will provide a great opportunity for identifying the molecular determinants for TRPA1 antagonists and developing of new analgesics as well as anti-inflammatory drugs targeting TRPA1.

## Materials and Methods

### Two-electrode Voltage Clamp Method

Chimeras and mutants for fTRPA1, hTRPA1, zebrafish (z) TRPA1a, and zTRPA1b, were heterologously expressed in *X. laevis* oocytes. Mature *X. laevis* females were purchased from Hamamatsu Seibutsu Kyozai (Hamamatsu, Japan) and reared at ~18 °C. Oocytes were surgically excised from anesthetized *X. laevis*, and follicular membranes were enzymatically removed from oocytes and ionic currents were recorded using the two-electrode voltage clamp method described previously^33^. 50 nl of complementary RNA (cRNA; 50–150 ng/μl) for fTRPA1, hTRPA1, zTRPA1a, or zTRPA1b chimeras (or mutants) was injected into defolliculated oocytes kept at 17 °C in MBSH-PS solution and ionic currents were recorded 2–5 days post-injection. Oocytes were voltage-clamped at −60 mV. All chemicals were diluted in ND96 bath solution containing 96 mM NaCl, 2 mM KCl, 1.8 mM CaCl_2_, 1 mM MgCl_2_, 5 mM Hepes, pH 7.4 (with NaOH), and applied to the oocytes by perfusion. ND96 Ca^2+^ free solution was prepared by removing 1.8 mM CaCl_2_ from the solution. The zTRPA1a and zTRPA1b expression vectors were kindly provided by Dr. Prober^34^. All animal care and experimental procedures were performed according to the National Institutes of Health and National Institute for Physiological Sciences guidelines (#16A075).

### Molecular biology experiments

We used expression vectors for wild type fTRPA1, hTRPA1, zTRPA1a, and zTRPA1b, all of which were expressed in either human embryonic kidney (HEK) 293 cells and/or in *Xenopus laevis* oocytes as described previously^33^. All single and double amino acid mutants for hTRPA1, fTRPA1 and zTRPA1b were generated using the PrimeSTAR Mutagenesis Basal kit (Takara) or QuickChange Site-Directed Mutagenesis kit (Stratagene) with some modification using specific primers (Supplementary Tables S2 and S3). The entire coding region of all TRPA1 mutant clones was verified by sequencing using the BigDye Terminator version 3.1 cycle sequencing kit (Applied Biosystems).

Full length chimeric TRPA1 cDNA was amplified by polymerase chain reaction (PCR). In the first step, different DNA fragments of TRPA1, F-H (T1-Ct) [TRPA1 (715–1119)], F-H (T1-T6)-F [hTRPA1(715–975)], F-H (T3-Ct) [hTRPA1(789–1119)], F-H (T5-Ct) [hTRPA1(844–1119)], and F-H (Ct) [hTRPA1(975–1119)] were amplified by PCR with an expression vector containing human TRPA1 cDNA as a template using the combination of primers listed in Supplementary Tables S2 and S3. DNA fragments of TRPA1 cDNA, fTRPA1(1–718) for F-H (T1-Ct), fTRPA1(1–718, 1001-1144) for F-H (T1-Ct)-F, fTRPA1(1–811) for F-H (T3-Ct), fTRPA1(1–867) for F-H (T5-Ct), and fTRPA1(1–999) for F-H (Ct) were also amplified by PCR with an expression vector containing TRPA1 as a template and the combination of primers listed in Supplementary Tables S2 and S3. In the second step, overlap extension PCR was performed with a mixture of these DNA fragments to amplify the chimeric TRPA1 cDNA which was then cloned using standard procedures.

### Intracellular Ca^2+^ imaging experiments

The procedure for Ca^2+^ imaging was described previously^10^. Briefly, the pcDNA3.1 (+) vectors containing hTRPA1 and hTRPA1-N885S were transfected into HEK293 cells using Lipofectamine reagent (Invitrogen) as per manufacturer’s instructions. Cells were used for [Ca^2+^]_i_ imaging experiments after incubation for approximately 24 h. Fura-2 was loaded into the cells by incubating at 37 °C for 0.5–1 h with fura-2 acetoxymethyl ester (5 μM) in D-MEM (high glucose, Wako) containing heat-denatured 10% FBS (Gibco), 0.5% penicillin/streptomycin (Invitrogen) and 1% GlutaMax (Invitrogen). Cells were transferred to recording chambers mounted on the stage of an inverted microscope equipped with an image acquisition and analysis system. All chemicals were applied by perfusion of bath solution.

To measure [Ca^2+^]_*i*_, cells were illuminated every 3 s with light at 340 and 380 nm. The intensity of the fluorescent signals at 500 nm emitted by light, excited at 340 (F340) and 380 (F380) nm were recorded, and their ratios (F340/F380) were calculated. Cells were continuously superfused with bath solution, and drugs were applied through the same tube. All experiments were carried out at room temperature (~25 °C).

### Chemicals

Cinnamaldehyde, HC-030031 and A-967079 were purchased from Wako, Sigma, and Santa Cruz Biotechnology respectively. All chemicals were dissolved in DMSO as stock solutions (0.1–2 M).

### Molecular dynamics simulation

We prepared the initial hTRPA1conformation for the molecular dynamics (MD) simulations employing the PDB structure (ID: 3J9P). Because several amino acid residues were missing in the PDB structure, we complemented the residues 664–679, 748–763, 786–802, and 1007–1030 using the modeling program MODELLER^35^,^36^. We then utilized the docking program AutoDock^37^ to obtain the docking conformation of HC with TRPA1-N855. The N- and C-terminal residues (up to residue 618 and after residue 1048) were removed to perform the MD simulations. By replacing N855 of this conformation with a serine, the initial conformation of the N855S mutant was prepared. To replace the asparagine with the serine, we removed the N_δ_ and H_δ_ atoms of the asparagine and replaced the C_γ_ atom and the O_δ_ atom with the O_γ_ atom and the H_γ_ atom, respectively.

HC is a non-standard organic molecule for MD simulation of biomolecules. We therefore determined the parameters for calculating the potential energy and force of HC. To derive the partial atomic charge of HC, we followed the RESP charge method^38^ and quantum chemical calculations were performed using the Gaussian09 program. Structure optimization of HC and electrostatic potential calculations were carried out using the Hartree-Fock level of theory with a 6–31 G (d) basis set. We used parameters of the general Amber force field except for the atomic partial charges^39^.

The Generalized-Ensemble Molecular Biophysics (GEMB) program developed by one of the authors (H.O.)^40^,^41^,^42^ was used to perform the MD simulations. The AMBER parm99SB force field^43^ was used for the TRP channel and the general AMBER force field was used for HC^39^. A cubic simulation box was employed with periodic boundary conditions. Electrostatic potential was calculated using the particle-mesh Ewald (PME) method^44^. The cut-off distance was 12 Å for the Lennard-Jones potential. Temperature was controlled at 298 K using the Nosé-Hoover thermostat^45^,^46^,^47^. Reversible multiple time-step MD techniques were applied^48^. The time-step was taken to be *Δt* = 0.5 fs for the bonding interactions and *Δt* = 2.0 fs for the non-bonding interactions. To maintain the atomic structure of the TRP channel in a vacuum, N, C_α_, and C atoms of the residues that form α-helix structures were restrained with a harmonic potential.

### Data Analysis

Data for the Ca^2+^ imaging experiments were obtained from at least three independent transfections. The data from current recordings were obtained from oocytes collected from at least three different frogs. The data are presented as the mean ± SEM (n = number of observations). Statistical analyses were tested using a one-way ANOVA followed by the Tukey-Kramer test with *p* values < 0.05. EC_50_ was determined using Origin software (OriginLab). The amino acid sequences of TRPA1 from the various vertebrate species were aligned using ClustalW implemented in MEGA5 and the amino acid similarities were calculated^49^,^50^.

## Additional Information

**How to cite this article**: Gupta, R. *et al*. Structural basis of TRPA1 inhibition by HC-030031 utilizing species-specific differences. *Sci. Rep.* **6**, 37460; doi: 10.1038/srep37460 (2016).

**Publisher's note:** Springer Nature remains neutral with regard to jurisdictional claims in published maps and institutional affiliations.

## Supplementary Material

Supplementary Information

Supplementary Movie 1
