# Supplementary material for: Structural basis of TRPA1 inhibition by HC-030031 utilizing species-specific differences
Source: Sci Rep. 2016 Nov 22;6:37460. doi: 10.1038/srep37460 (PMC5118716; doi:10.1038/srep37460)
Supplement: Supplementary Information [file srep37460-s1.pdf]

Title:

Structural basis of TRPA1 inhibition by HC-030031 utilizing species-specific differences

Abbreviated title:

Structural basis of TRPA1 inhibition by HC-030031

Author names and affiliation:

Rupali Gupta[1, 2], Shigeru Saito*[1,2] , Yoshiharu Mori[3], Satoru G. Itoh[3,4], Hisashi Okumura[3,4], Makoto Tominaga*[1,2]

[1]Division of Cell Signaling, Okazaki Institute for Integrative Bioscience (National Institute for Physiological Sciences), National Institutes of Natural Sciences, Okazaki, Japan. [2]Department of Physiological Sciences, SOKENDAI (The Graduate University for Advanced Studies), Okazaki, Japan. [3]Research Center for Computational Science, Institute for Molecular Science, Okazaki, Japan. [4]Department of Structural Molecular Science, SOKENDAI (The Graduate University for Advanced Studies), Okazaki, Japan

Movie 1. The molecular dynamics simulation: highlighting the stabilized fluctuation between N855 of the hTRPA1 channel and HC.

Figure S1. (a) Species specific antagonistic effects of A-967079, AP-18 and HC-030031 on TRPA1 from different species and (b) their structural differences. *One report showed that A96 activates frog and chicken TRPA1 at higher concentration [12].

Figure S2. (a) Dose-dependent inhibition of wt-hTRPA1 by HC. Each bar represents the mean ± SEM. (**P<0.01), one way ANOVA post hoc Tukey test. wt-hTRPA1 (CA 0.1 µM, n=4; CA + 10 µM HC, n=4; CA + 25 µM HC, n=4; CA + 50 µM HC, n=4). (b-c) Representative traces of wt-hTRPA1 (b) or wt-fTRPA1 (c) currents in response to CA application in *X. laevis* oocytes in the absence of extracellular Ca^{2+}. (d-e) Representative traces showing that species-specific difference of wt-hTRPA1 (d) or wt-fTRPA1 (e) currents in response to non-electrophilic compound carvacrol (Carv) 0.5 and 2 mM for wt-hTRPA1 and wt-fTRPA1, respectively in the presence or absence of 50 µM HC.

Figure S3. Dose-dependent profiles of the CA-evoked currents of chimeric channels between wt-fTRPA1 and wt-hTRPA1. (a) F-H (T1-Ct), (b) F-H (T1-T6), (c) F-H (T3-Ct), (d) F-H (T5-Ct), and (e) F-H (Ct).

Figure S4. Representative traces showing that 50 μM HC inhibited the CA-evoked currents in F-H (T1-Ct) (a), F-H (T1-T6)-F (b), F-H (T3-Ct) (c), F-H (T5-Ct) (d) and F-H (Ct) (e).

Figure S5. (a) A representative trace of the hTRPA1-N855S currents in response to repeated CA application. (b-c) Representative traces of the CA-evoked currents in wt-hTRPA1 or hTRPA1-N855S in the presence of 50 μM HC (b) or 1 μM A96 (c and d).

Figure S6. (a, b) CA stimulation increased $[Ca^{2+}]_i$ in a dose-dependent manner in HEK293T cells expressing either wt-hTRPA1 (a) or hTRPA1-N855S (b). (c, d) CA (0.1 mM)-evoked increase in $[Ca^{2+}]_i$ in HEK293 cells expressing wt-hTRPA1 or hTRPA1-N855S. Each bar represents the mean ± SEM. (c) Comparison of the normalized ratio between wt-hTRPA1 (n=5) and hTRPA1-N855S (n=7) from (c) and (b).

Figure S7. (a-b) zTRPA1a and zTRPA1b exhibited different sensitivities to CA at different concentrations. (c-d) Representative traces of CA-evoked currents in *X. laevis* oocytes expressing wt-zTRPA1a or wt-zTRPA1b.

Figure S8. (a, b) Representative traces of CA-evoked currents in *X. laevis* oocytes expressing hTRPA1-N855R (a) or zTRPA1b-R860N (b). (c-f) Effects of 50 μM HC (c,

d) or 1 μM A96 (e, f) on CA-evoked currents in hTRPA1-N855R (c, e) or zTRPA1b-R860N (d, f).

Figure S9. (a) Snapshots of the MD simulation of the N855S mutant at 100 ns. The HC molecule and amino acid residue S855 are shown in a stick representation. These snapshots were rendered using the RasMol graphic software [51]. (b) A time series of the distance between the O atom of HC and the Hγ atom of S855 in the MD simulation.

Supplementary Table S1: Effect of 50μM HC on different hTRPA1 mutants and WT

**WT & Mutants**	**(CA+50 μM HC)/(CA)**
wt-fTRPA1	1.12
N855S	0.26
V876L	0.13
L848I	0.1
E924K	0.06
L931I	0.05
L902F-S903A	0.03
M844V	0.03
L935F	0.02
K969R	0.02
H970N	0.01
R919E	0.01
wt-hTRPA1	0.01
L890F-N892Y	0.01
T908S	0
S900R	0
N929D	0
Q940H	0
Q1000K	0
E1097D	0
L1045W-Y1049N	0
L1107A	0

Effects of repeated application of CA alone or with 50 μM HC on different hTRPA1 mutants are summarized. Currents in CA + 50μM HC (average normalized currents for CA with HC) were divided by average normalized current for CA alone. For all mutants n=3-7.

Supplementary Table S2:

(a) Sequences of primers used for construction of chimeras between hTRPA1 and fTRPA1.

Chimeras	Primers
frog TRPA1-F-KpnI	TCA TAA GGT ACC ATG AGG AGG TCG ATC AGA AG
F-H (T1-Ct)-R	ATG AGC TCT AAA TCC ATA GGC CAT CCA TTT CAT AAG C
F-H (T1-Ct)- F	GAA ATG GAT GGC CTA TGG ATT TAG AGC TCA TAT GAT GAA T
human TRPA1-R-NotI	TCA TAA GCG GCC GCCTAA GGC TCA AGA TGG TGT G
F-H-(T1-T6)-F-Fwd	TTG TAT CTC CAA AGG TTT GAA AGT TAT G
frog TRPA1-NotI-pOX-R	TAG ACT CGA GCG GCC GCCTAA ACG TTC TTA CAG GAT TTT ACA G
F-H (T3-Ct)-F	AGA GGC GGG GCA AAT TTT CCA ACA G
F-H (T3-Ct)-R	ATT TGC CCC GCC TCT TTT ACA ATT CCA AAT AGA CTC
F-H (T5-Ct)-F	GTT TGC ATC TTG GAT GAA TTT CTT ATT GTA TCT TCA AAG
F-H (T5-Ct)-R	ATC CAA GAT GCA AAC ACA GCA A
F-H (Ct)-F	GAA AAG GAT AGC TAT GCA GGT GGA ACT TC
F-H (Ct)-R	ATA GCT ATC CTT TTC AAG CAT GCA TTT CG

(b) Sequences of primers used for construction of fTRPA1 and zTRPA1b mutants.

Reverse-mutants	Primers
Frog-TRPA1 S880N-F	CTC CAA AGG TTT GAA AAT TAT GG
Frog-TRPA1 S880N-R	CCA TAA TTT TCA AAC CTT TGG AG
Zebrafish-TRPA1b R860N-F	TTT GAG AAT ATT GGG ATT TAC GTG GTG
Zebrafish-TRPA1b R860N-R	CCC AAT ATT CTC AAA TCT CTG AAA GTA

Supplementary Table S3: Sequences of primers used for construction of hTRPA1 mutants

Mutant	Primers
M844V-F	GCT GTT TAC TTC TAT TGG GTT AAT TTC TTA TTG
M844V-R	CAA TAA GAA ATT AAC CCA ATA GAA GTA AAC AGC
L848I-F	CTT CTA TTG GAT GAA TTT CTT AAT TTA TCT TCA AAG
L848I-R	CTT TGA AGA TAA ATT AAG AAA TTC ATC CAA TAG AAG
N855S-F	GTA TCT TCA AAG ATT TGA AAG TTG TG
N855S-R	CAC AAC TTT CAA ATC TTT GAA GAT AC
V876L-F	GTT GAG GTC TAC AGT TCT GTT TAT CTT CCT TC
V876L-R	GAA GGA AGA TAA ACA GAA CTG TAG ACC TCA AC
L890F-N892Y-F	CTC AGC TTT TAC ATC TTT CTG TAT TTA CAG GAT CCC TTC
L890F-N892Y-R	GAA GGG ATC CTG TAA ATA CAG AAA GAT GTA AAA GCT GAG
S900R-F	CAG GAT CCC TTC AGC AGG CCA TTG C
S900R-R	GCA ATG GCC TGC TGA AGG GAT CCT G
L902F-S903A-F	GCT CTC CAT TGT TTG CCA TAA TCC AGA CCT TCA GC
L902F-S903A-R	GCT GAA GGT CTG GAT TAT GGC AAA CAA TGG AGA GC
T908S-F	GCT TTC TAT AAT CCA GTC ATT CAG CAT G
T908S-R	CAT GCT GAA TGA CTG GAT TAT AGA AAG C
R919E-F	GGA GAT ATC AAT TAT GAA GAG TCC TTC CTA GAA CC
R919E-R	GGT TCT AGG AAG GAC TCT TCA TAA TTG ATA TCT CC
E924K-F	CGA GAG TCC TTC CTA AAA CCA TAT CTG AGA AAT G
E924K-R	CAT TTC TCA GAT ATG GTT TTA GGA AGG ACT CTC G
N929D-F	CCA TAT CTG AGA GAC GAA TTG GCA C
N929D-R	GTG CCA ATT CGT CTC TCA GAT ATG G
L931I-F	CTG AGA AAT GAA ATA GCA CAT CCA GTT C
L931I-R	GAA CTG GAT GTG CTA TTT CAT TTC TCA G
L935F-F	GGC ACA TCC AGT TTT TTC CTT TGC ACA AC
L935F-R	GTT GTG CAA AGG AAA AAACTG GAT GTG CC
Q940H-F	CTG TCC TTT GCA CAC CTT GTT TCC TTC
Q940H-R	GAA GGA AAC AAG GTG TGC AAA GGA CAG
K969R-F	GAC ATT GCT GAG GTC CAG GCA CAT GCA TCA TTG
K969R-R	CAA TGA TGC ATG TGC CTG GAC CTC AGC AAT GTC
H970N-F	GCT GAG GTC CAG AAA AAT GCA TCA TTG AAG
H970N-R	CTT CAA TGA TGC ATT TTT CTG GAC CTC AGC
Q1000K-F	CGC AAA GTG GAT AAG AAA TCC ACC
Q1000K-R	GGT GGA TTT CTT ATC CAC TTT GCG
L1040M-F	GCT GAT AAA TCT ATG GAA ATG G
L1040M-R	CCA TTT CCA TAG ATT TAT CAG C
L1045W-Y1049N-F	GAA ATG GAA ATA TGG AAG CAG AAA AAC CGG CTG AAG G
L1045W-Y1049N-R	CCT TCA GCC GGT TTT TCT GCT TCC ATA TTT CCA TTT C
L1057M-E1059Q-F	GGA TCT TAC TTT TAT GCT GCA GAA ACA GCA TGA GC
L1057M-E1059Q-R	GCT CAT GCT GTT TCT GCA GCATAA AAG TAA GAT CC
F1087C-F1091C-F	GCC ATT GTT CTT GTC AAG ACA GGA TGA AGA AAG AGC
F1087C-F1091C-R	GCT CTT TCT TCA TCC TGT CTT GAC AAG AAC AAT GGC
E1097D-F	GAG CAG ATG GAT CAA AGG AAT AGC
E1097D-R	GCT ATT CCT TTG ATC CAT CTG CTC
L1107A-F	GGA ATA CTG TGG GGA GAG CAG TCA AGG C
L1107A-R	GCC TTG ACT GCT CTC CCC ACA GTA TTC C

Figure S1

a

Species	A-967079 & AP-18	HC-030031
Frog TRPA1	*No Inhibition	No Inhibition
Human TRPA1	Inhibition	Inhibition
Mouse TRPA1	Inhibition	Inhibition
Green anole TRPA1	No Inhibition	Inhibition
Chicken TRPA1	*No Inhibition	Inhibition

b

A-967079

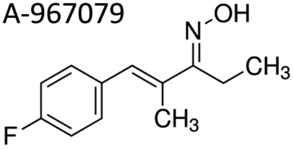

AP-18

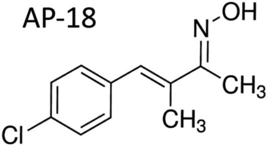

HC-030031

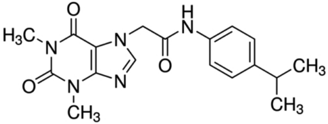

Figure S2

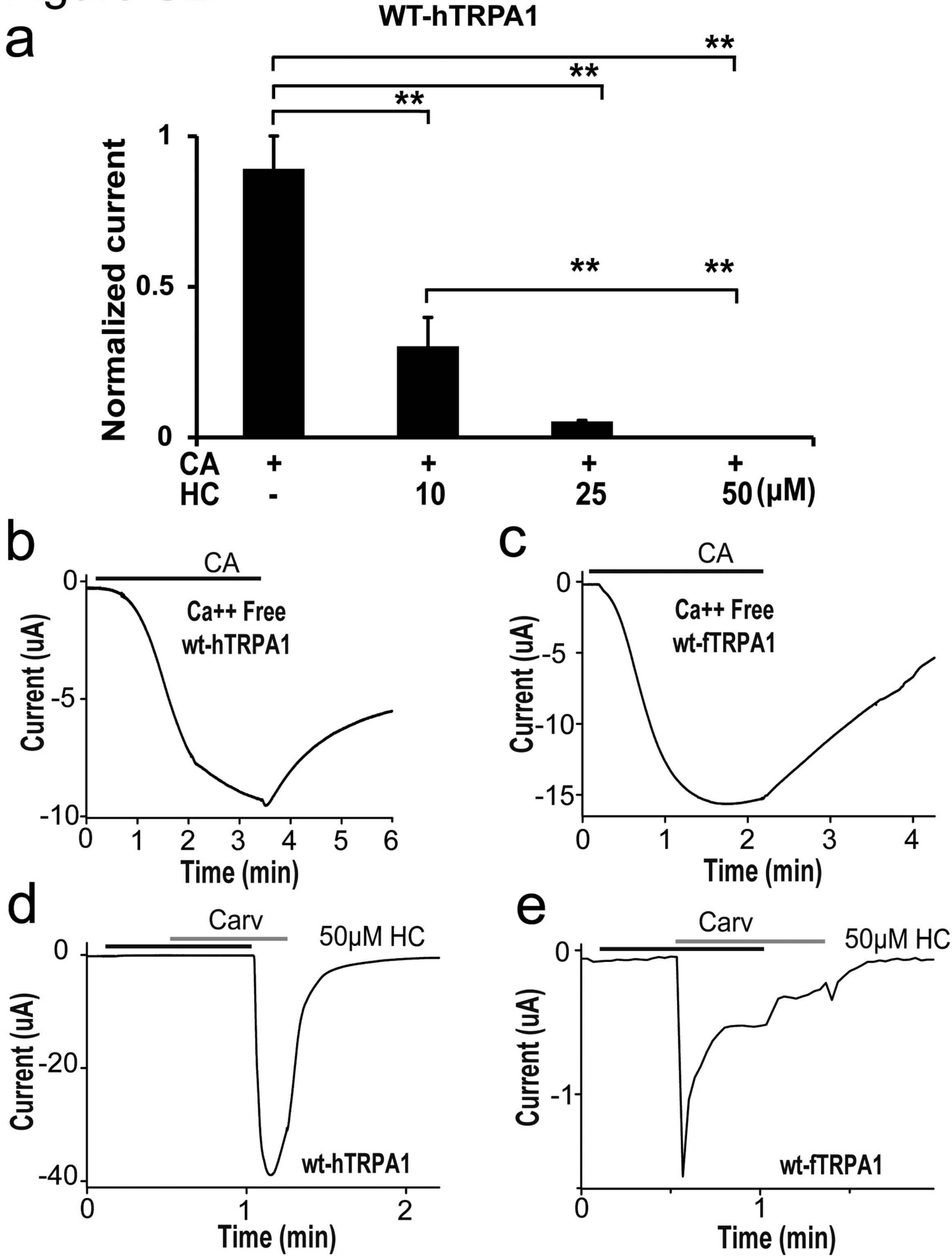

a
WT-hTRPA1
**
**
**
**
**
Normalized current
1
0.5
0
CA + + + +
HC - 10 25 50 (μM)
b
CA
Ca++ Free
wt-hTRPA1
Current (uA)
0
-5
-10
0 1 2 3 4 5 6
Time (min)
c
CA
Ca++ Free
wt-fTRPA1
Current (uA)
0
-5
-10
-15
0 1 2 3 4
Time (min)
d
Carv
50μM HC
wt-hTRPA1
Current (uA)
0
-20
-40
0 1 2
Time (min)
e
Carv
50μM HC
wt-fTRPA1
Current (uA)
0
-1
0 1
Time (min)

Figure S3

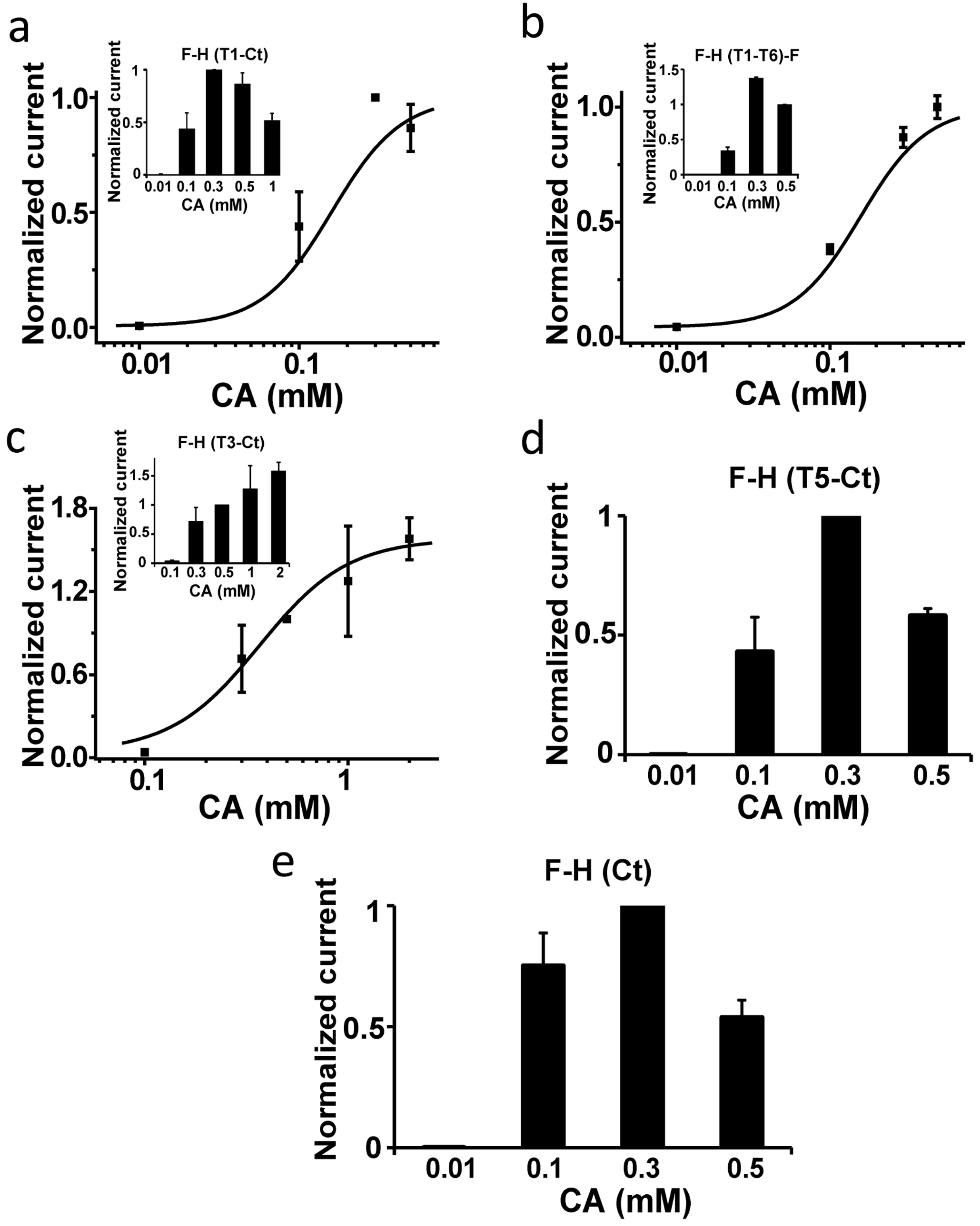
a
F-H (T1-Ct)
Normalized current
CA (mM)
b
F-H (T1-T6)-F
Normalized current
CA (mM)
c
F-H (T3-Ct)
Normalized current
CA (mM)
d
F-H (T5-Ct)
Normalized current
CA (mM)
e
F-H (Ct)
Normalized current
CA (mM)

Figure S4

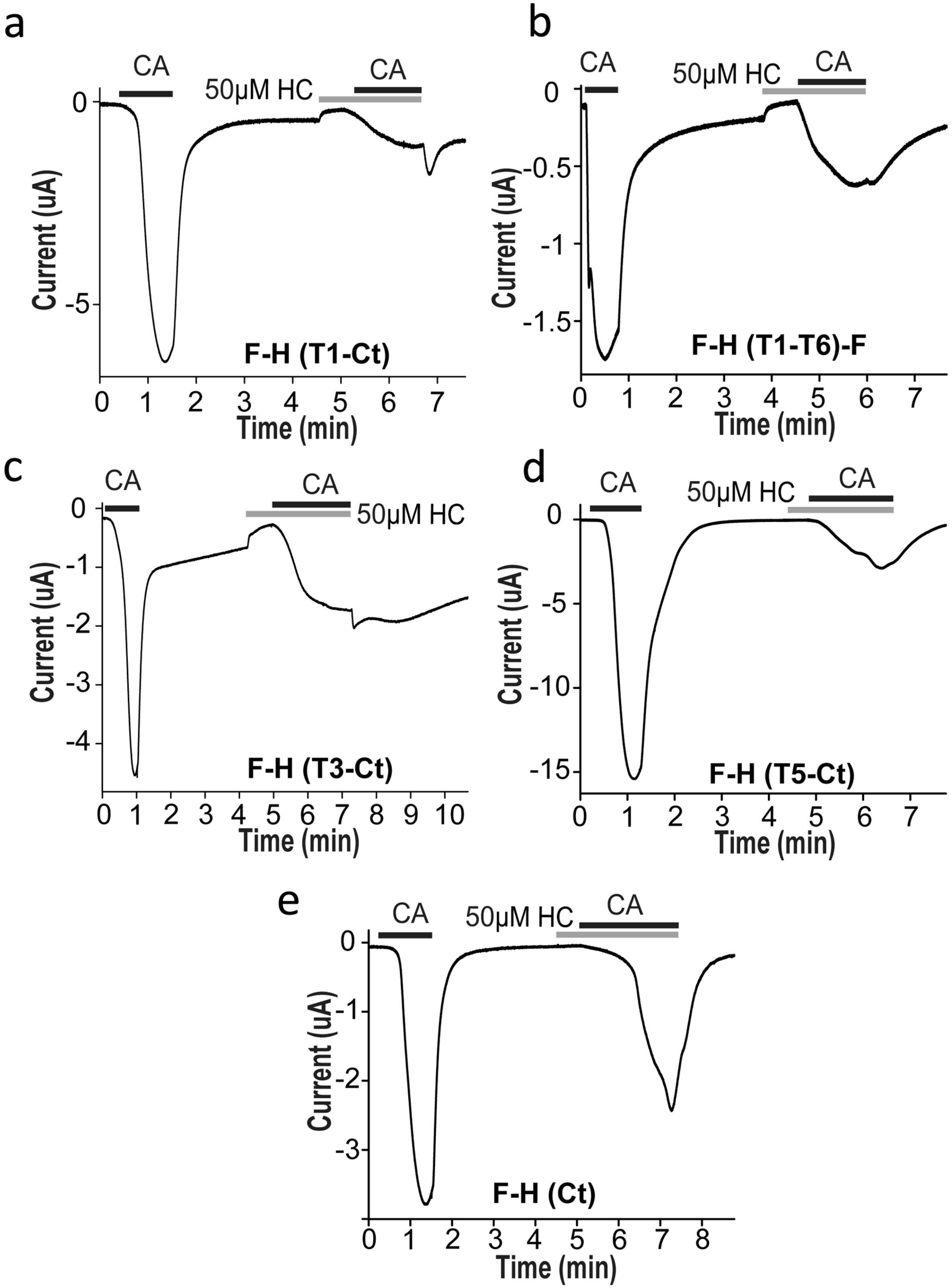

a
CA
50µM HC
CA
F-H (T1-Ct)
Current (uA)
Time (min)
b
CA
50µM HC
CA
F-H (T1-T6)-F
Current (uA)
Time (min)
c
CA
CA
50µM HC
F-H (T3-Ct)
Current (uA)
Time (min)
d
CA
50µM HC
CA
F-H (T5-Ct)
Current (uA)
Time (min)
e
CA
50µM HC
CA
F-H (Ct)
Current (uA)
Time (min)

Figure S5

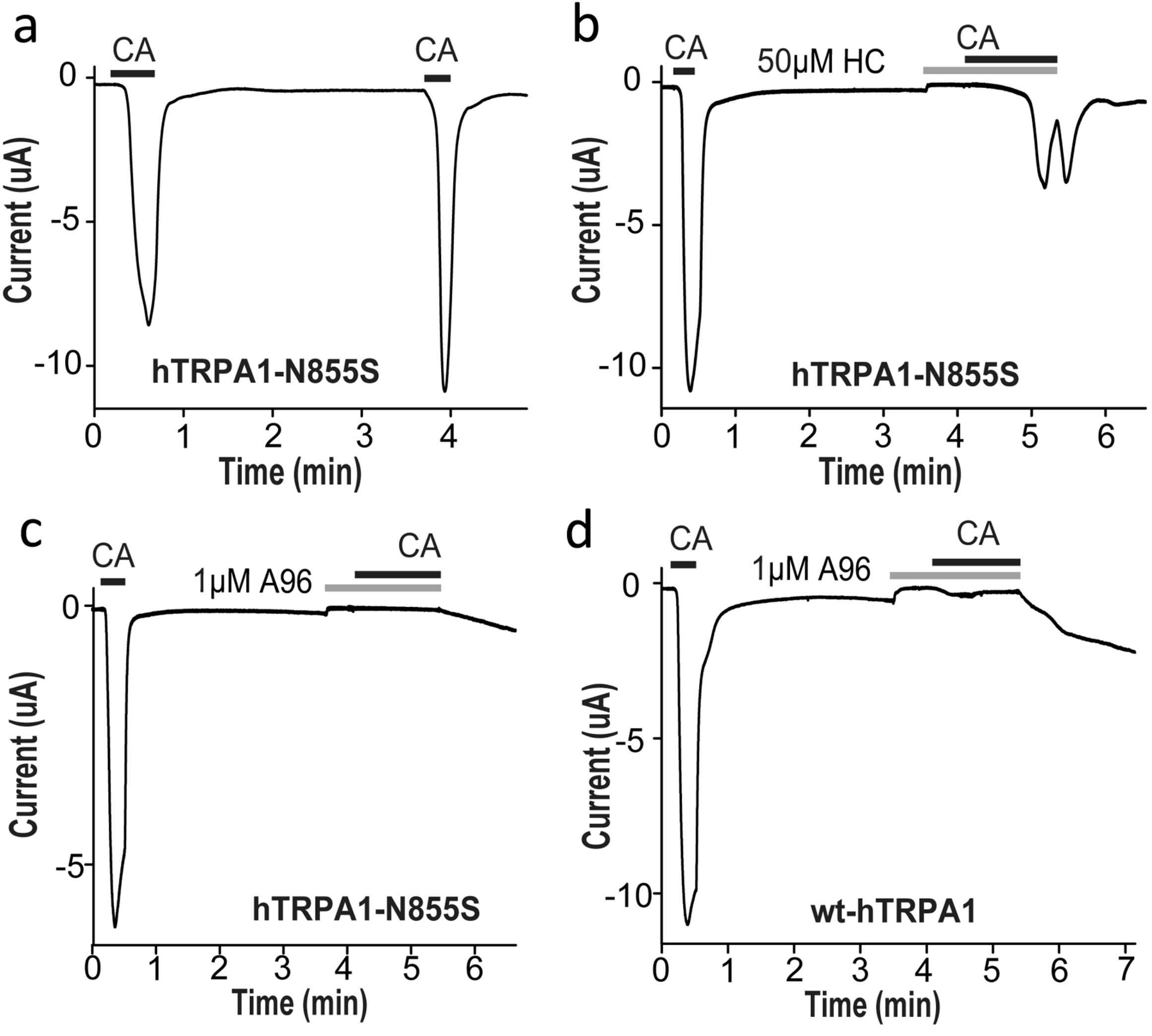

a
CA
CA
0
-5
-10
Current (uA)
hTRPA1-N855S
0
1
2
3
4
Time (min)
b
CA
50μM HC
CA
0
-5
-10
Current (uA)
hTRPA1-N855S
0
1
2
3
4
5
6
Time (min)
c
CA
1μM A96
CA
0
-5
Current (uA)
hTRPA1-N855S
0
1
2
3
4
5
6
Time (min)
d
CA
1μM A96
CA
0
-5
-10
Current (uA)
wt-hTRPA1
0
1
2
3
4
5
6
7
Time (min)

Figure S6

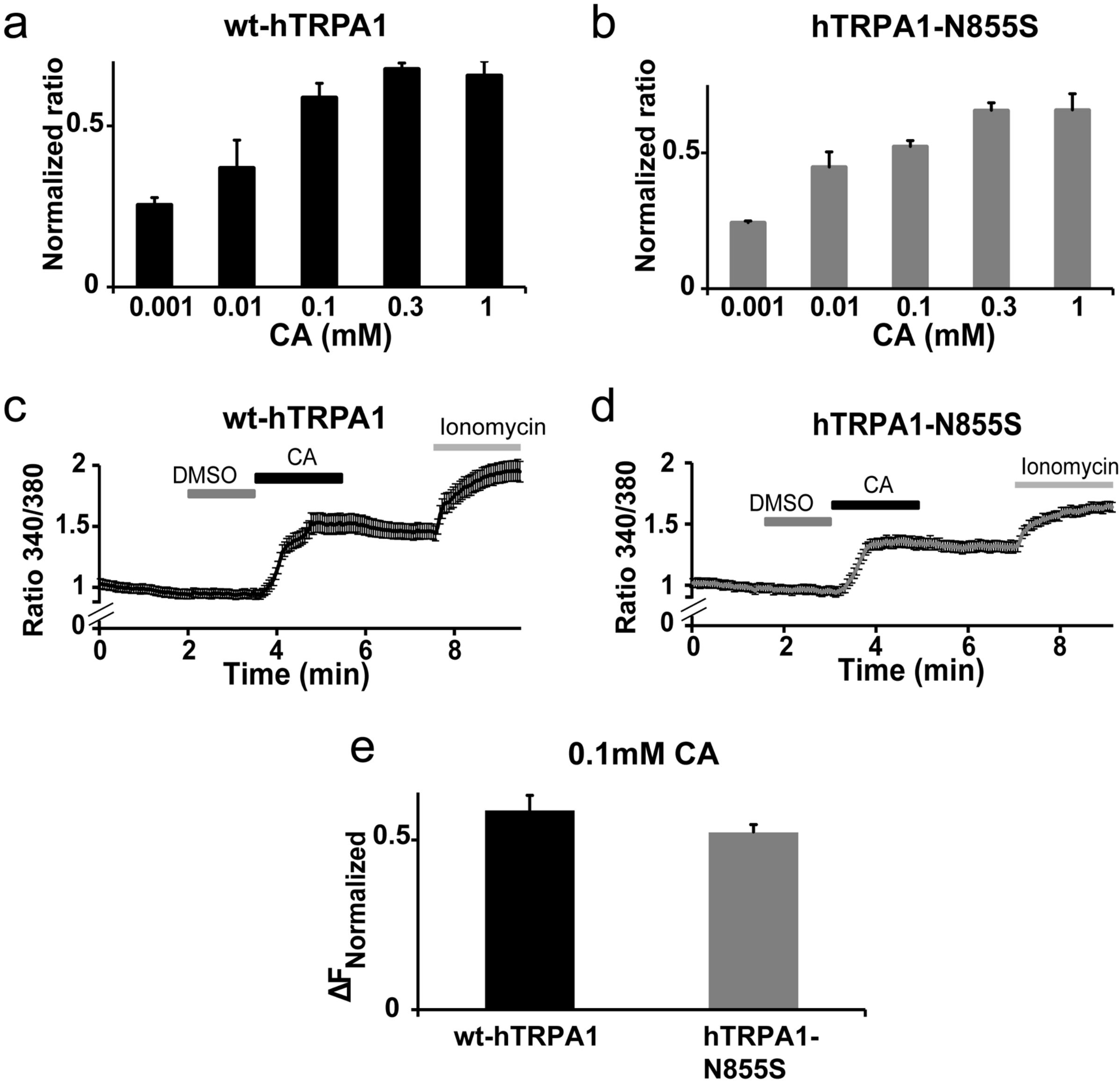
a
wt-hTRPA1
Normalized ratio
0.5
0
0.001
0.01
0.1
0.3
1
CA (mM)
b
hTRPA1-N855S
Normalized ratio
0.5
0
0.001
0.01
0.1
0.3
1
CA (mM)
c
wt-hTRPA1
Ionomycin
CA
DMSO
Ratio 340/380
2
1.5
1
0
0
2
4
6
8
Time (min)
d
hTRPA1-N855S
Ionomycin
CA
DMSO
Ratio 340/380
2
1.5
1
0
0
2
4
6
8
Time (min)
e
0.1mM CA
ΔF Normalized
0.5
0
wt-hTRPA1
hTRPA1-
N855S

Figure S7

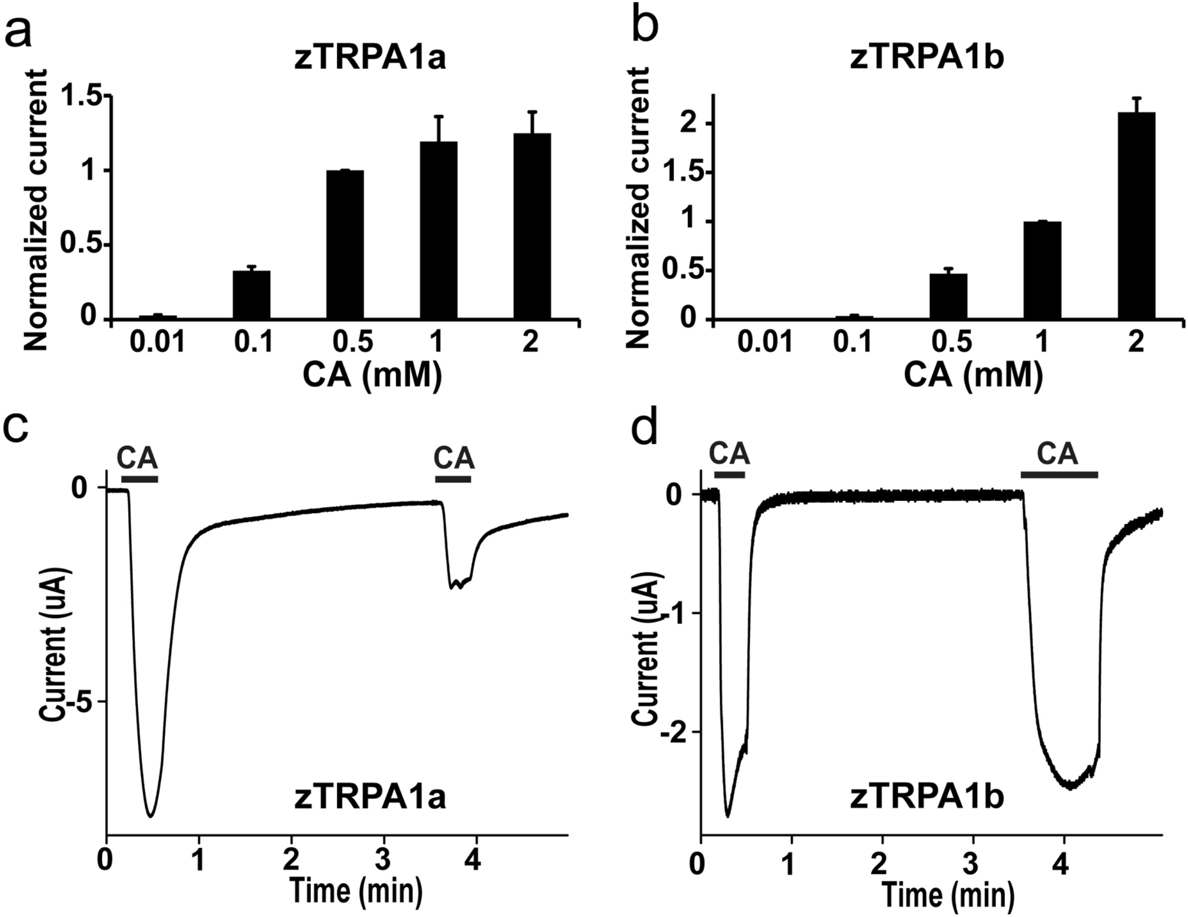

a
zTRPA1a
Normalized current
1.5
1
0.5
0
0.01
0.1
0.5
1
2
CA (mM)
b
zTRPA1b
Normalized current
2
1.5
1
0.5
0
0.01
0.1
0.5
1
2
CA (mM)
c
CA
CA
0
-5
Current (uA)
zTRPA1a
0
1
2
3
4
Time (min)
d
CA
CA
0
-1
-2
Current (uA)
zTRPA1b
0
1
2
3
4
Time (min)

Figure S8

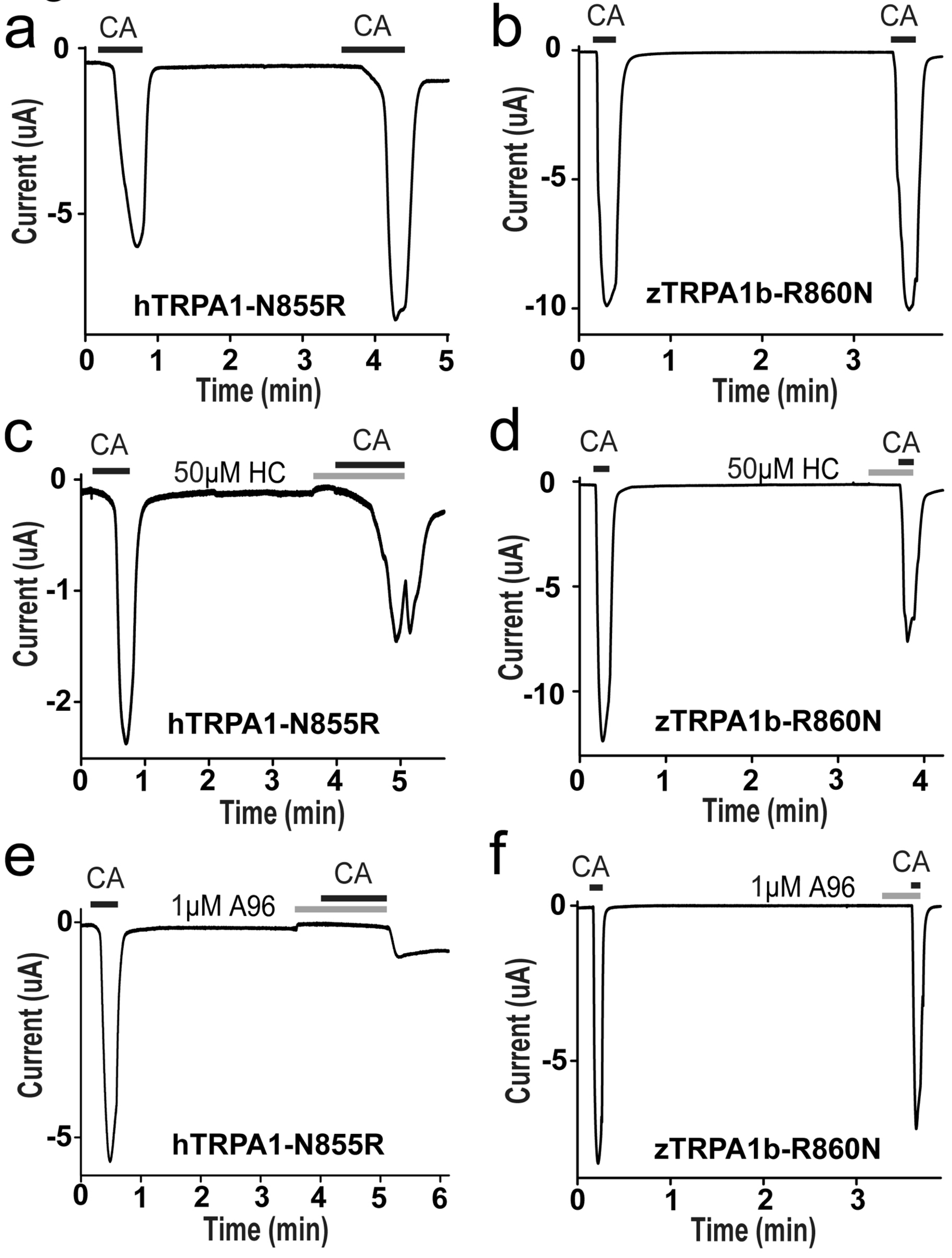

a
CA
CA
0
-5
Current (uA)
hTRPA1-N855R
0 1 2 3 4 5
Time (min)
b
CA
CA
0
-5
-10
Current (uA)
zTRPA1b-R860N
0 1 2 3
Time (min)
c
CA
50μM HC
CA
0
-1
-2
Current (uA)
hTRPA1-N855R
0 1 2 3 4 5
Time (min)
d
CA
50μM HC
CA
0
-5
-10
Current (uA)
zTRPA1b-R860N
0 1 2 3 4
Time (min)
e
CA
1μM A96
CA
0
-5
Current (uA)
hTRPA1-N855R
0 1 2 3 4 5 6
Time (min)
f
CA
1μM A96
CA
0
-5
Current (uA)
zTRPA1b-R860N
0 1 2 3
Time (min)

Figure S9

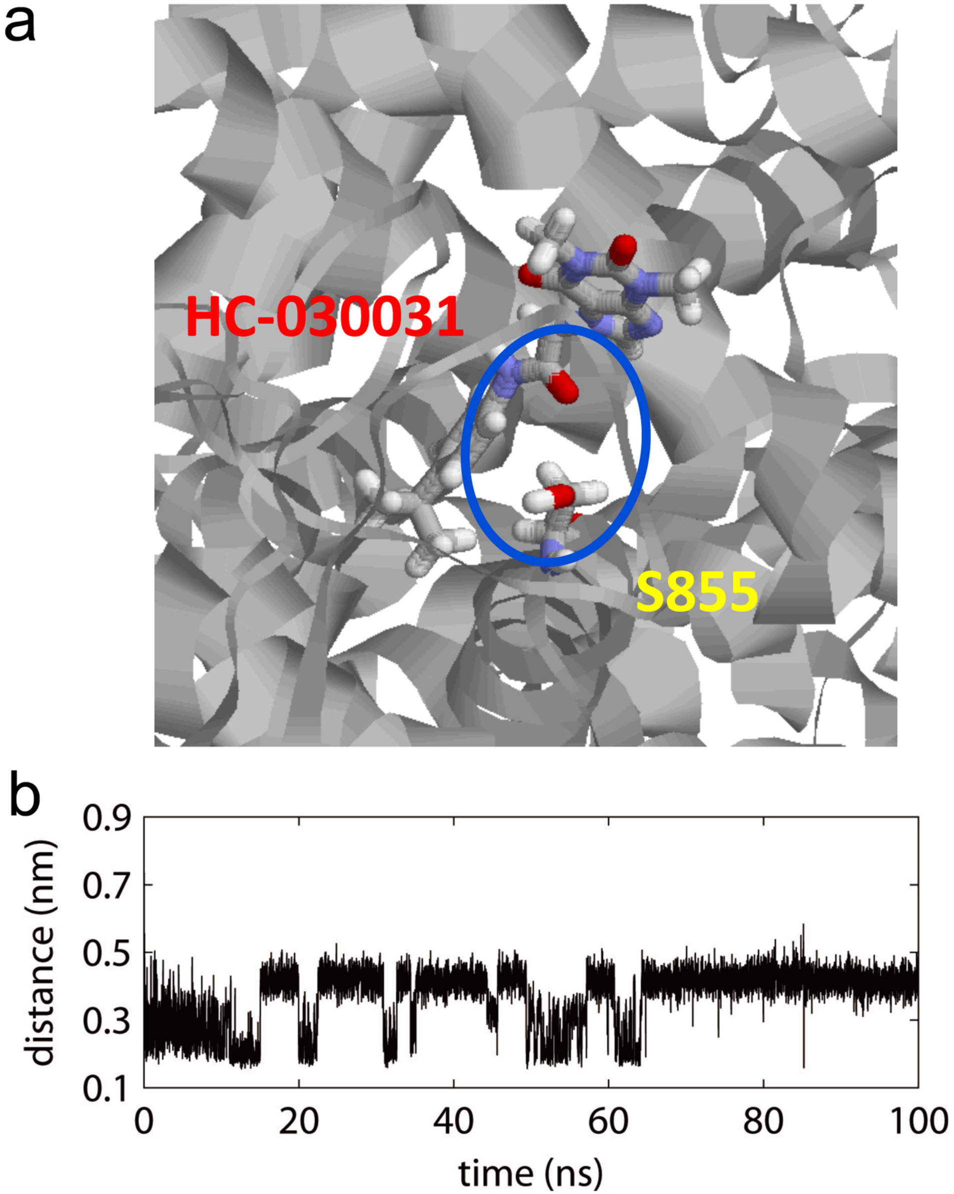
a
HC-030031
S855
b
0.9
0.7
0.5
0.3
0.1
distance (nm)
0
20
40
60
80
100
time (ns)
